# Definitions and Assessment Methods of ‘Home Cooking’ in Studies with Dietary Variables: A Scoping Review

**DOI:** 10.3390/nu14163344

**Published:** 2022-08-15

**Authors:** Xiaoyi Yuan, Aya Fujiwara, Mai Matsumoto, Ryoko Tajima, Chisa Shinsugi, Emiko Koshida, Hidemi Takimoto

**Affiliations:** 1 Department of Nutritional Epidemiology and Shokuiku, National Institutes of Biomedical Innovation, Health, and Nutrition, 1-23-1 Toyama, Shinjuku-ku, Tokyo 162-8636, Japan; 2 Department of Social and Preventive Epidemiology, School of Public Health, University of Tokyo, Bunkyo-ku, Tokyo 113-0033, Japan; 3 Department of Epidemiology and Prevention, Center for Clinical Sciences, National Center for Global Health and Medicine, 1-21-1 Toyama, Shinjuku-ku, Tokyo 162-8655, Japan

**Keywords:** home cooking, food preparation, diet, intake, methodology, dietary assessment

## Abstract

Home cooking is a complex idea that involves multiple skills and behaviors and can be interpreted differently. Using six databases (two of which were Japanese), this scoping review examined the definitions and methods used in studies investigating the relationship between home cooking and dietary variables. Of the 40 studies (2 in Japanese) included in this review, 8 provided definitions but did not specify the extent or level that convenience foods can be allowed in food preparation. The methods were classified into two categories, namely, perception-dependent (*n* = 29) if using a self-reported instrument, or perception-independent (*n* = 11) if based on investigators’ classification. Subsequently, indicators of home cooking were classified based on survey attributes (e.g., frequency, location). All but five studies used single indicators, primarily the preparation frequency (*n* = 18). Quality of analysis was also evaluated. Studies that used multiple indicators or perception-independent methods showed high or moderate overall quality. In contrast, studies that used single indicators based on perception-dependent methods tended to have a low overall quality. The consistency of the relationship between home cooking and dietary variables depended on study quality. In conclusion, the definitions of home cooking were inconsistent across studies, and lacked consensus for examining the association between dietary outcomes.

## 1. Introduction

Diet is a modifiable risk factor for non-communicable diseases [1]. The food industry has been providing various food products and meal options (e.g., takeaway, ready-to-eat, and restaurant meals) to satisfy people’s needs for convenience [2,3,4]. However, these foods have been reported to contain high energy, saturated fatty acids (SFA), and salt [5,6,7,8,9]. Home cooking (HC), to some extent, ensures greater control over food choices [10,11], which makes it a potential target for public health interventions and policy developments to promote healthy eating [12,13,14]. The relationship between HC and dietary variables (e.g., dietary intake and diet quality) has been explored in several studies, but with inconsistent findings [15,16,17,18,19,20,21]. For example, some studies have shown that HC might be positively related to higher fruit and vegetable intake [16,17], diet quality [16,18], and greater adherence to dietary reference intakes [19]. However, other studies have reported null relationships [15,20,21].

Certainly, HC is not synonymous with healthy cooking, as initiating the cooking behaviors may not be driven by health consciousness [22]. However, these inconsistent findings may also be partially explained by the lack of a consensual definition and the complex nature of HC [23,24]. Without a specific definition of HC based on what investigators intend to assess, the results of HC assessments may depend on participants’ perceptions; for example, both “cooking from scratch” and “microwaving frozen meal” can be perceived as HC [25,26]. These perceptions may differ according to one’s food environment, culture, time and monetary constraints, personal values regarding foods and health, and so on [23,24,25,26,27,28,29,30,31]. Moreover, an HC assessment can be performed using various indicators [24]. For instance, HC can be assessed based on the frequency of food preparation [20] or food consumption [16], levels of food preparation [17], or the location of obtaining food [32]. Measurements with different indicators may also differ in accuracy and precision, thereby contributing to inconsistent results.

A few reviews have focused on the determinants of HC with varied outcomes (e.g., dietary intake, social relationships, and health status) [33,34], but not specifically on the definitions and methods used for assessing HC. In addition, the studies included in these reviews were mainly conducted in Western countries. The findings may therefore be limited in their generalizability to other cultures with different food environments, food and cooking values, and food-preparation techniques (e.g., Japan) [27,35,36,37]. A scoping review is considered appropriate for identifying the definitions and methods for assessing HC and identifying research gaps in this domain [38]. This scoping review included studies published in English and Japanese with the primary objective of providing an overview of the definitions and assessment methods of HC and the relationship between HC and dietary variables. The secondary objective was to identify research gaps in the current literature regarding HC and dietary variables, thereby providing implications for future research.

## 2. Materials and Methods

The review protocol was drafted based on the Preferred Reporting Items for Systematic Reviews and Meta-Analyses-Scoping Review Extension [39] and the guidelines developed by the Joanna Briggs Institute [38] and is available upon request.

### 2.1. Eligibility Criteria

Peer-reviewed original research studies were included if published in English or Japanese; they included independent human participants and examined either the association between HC (or home-cooked food products) and dietary variables (e.g., dietary intake and diet quality) or the contribution of a home-cooked food to dietary intake. In this study, the term HC refers to food preparation practices (regardless of the use of heat) in a household setting or in a setting attached to a kitchen that participants can access freely. Only studies aimed to examine the behaviors of food preparation practices (not assistance) and/or consumption were included. Studies targeting people with chronic diseases, pregnant women, or people on a special diet (e.g., a vegetarian diet) were not excluded, as the review aimed to identify definitions and methods rather than assessing the strength of the association between HC and dietary variables (Table S1 presents the detailed eligibility criteria).

### 2.2. Search Strategy

The PubMed, Web of Science, Scopus, and ProQuest databases, along with two Japanese databases, Ichūshi and CiNii, were used to identify the relevant articles. The search was supplemented by a manual search of the reference lists of the included studies. For searches undertaken in English, combinations of terms related to “cooking”, “diet”, and “home” was searched, with language filters for English and Japanese. For searches undertaken in Japanese, combinations of terms related to “home” (i.e., the characters for *ie* and *taku*), “cooking” (i.e., the Chinese characters for *ryōri* and *chōri*), and “eating” (or “food”; i.e., the character for *shoku* because it is shared by *taberu* (to eat) and *shokuhin* (food)) were searched. (File S1 presents the detailed search). There was no restriction on the publication date; the last search was conducted on 11 July 2021.

### 2.3. Selection of the Sources of Evidence

Records retrieved from all the databases were exported as Microsoft Excel files or files with comma-separated values that were imported into Microsoft Excel; duplicates were removed thereafter. All titles and abstracts retrieved from the databases were screened by one reviewer (X.Y.) for potential records; the reviewer (X.Y.) also conducted the full-text screening on potential records. Parallel screening was conducted by a review team (comprising A.F., M.M., R.T., C.S., and E.K.), each member was responsible for an equal proportion of the retained records. Disagreements were resolved by consensus or by consulting a third reviewer (H.T.) when needed. The excluded records and reasons for exclusion were recorded.

### 2.4. Data Extraction and Synthesis

The data were extracted using a standardized table specifically developed for this review. One reviewer (X.Y.) was responsible for extracting all the included studies. The extracted data were verified by the same review team (each member was responsible for an equal proportion of the studies included). 

The following information was extracted: first author, year of publication, country of study, participants’ characteristics, sample size, the definition of HC, method for assessing HC, dietary assessment methods, concurrence between meal occasions investigated for HC and dietary variables, data analysis method, and confounding controls. HC definitions were determined based on using expressions such as “defined as” or “an example of”, in association with the terms to be defined. Definitions were further classified and summarized into groups based on themes (if any could be extracted). In addition, based on the results of the HC survey, items depended on participants’ perceptions, and the methods for assessing HC were categorized as perception-dependent (e.g., self-reported questionnaire) or perception-independent (e.g., investigators’ classification based on information obtained from the diet record) methods. Unlike the perception-dependent method, which required participants to be informed of the definition, the perception-independent methods involved the extraction of the classification procedure details from the studies. The terms related to HC (e.g., home-cooked meals, food-prepared at home) used in the method were also extracted. Furthermore, HC indicators were identified based on the survey attribute (e.g., frequency of preparation, food preparation location). The results of the studies that investigated associations between HC and dietary variables were summarized based on whether there was a significantly positive, significantly negative, or null association with an increased magnitude of HC (e.g., increased frequency of preparation). The percentages were directly extracted for studies that assessed the contribution of HC to dietary intakes.

### 2.5. Quality Assessment of Analyses

Quality assessment was performed based on previously developed criteria [40,41] that were adapted to fit the purpose of this review. Six domains were assessed in this study. (1) The representativeness of the target population was assessed based on whether the study used a nationally representative sample, or the study’s response rate. (2) Confounding controls were assessed by adjusting for the potential covariates. Based on previous studies [22,42,43], age, sex, ethnicity (or race), education, income, employment, and household structure (or marital status or number of children) were included as potential covariates. (3) Methods for assessing HC were evaluated based on whether multiple indicators were applied; if not, whether a method was perception-dependent. (4) The instruments used for assessing dietary variables were analyzed based on whether a more detailed instrument (e.g., a 24-h dietary recall or diet record) or a validated instrument was used. (5) The concurrence between meal occasions was examined between those investigated for HC and dietary variables. (6) The data analysis was assessed based on the technique used, whether multivariate analysis or weights were applied to reflect national representativeness. Each domain was rated at one of the three levels (high, moderate, or low). However, concurrence and data analysis were considered complimentary items and rated at two levels (high or moderate), whereas the low rating level was weighted toward the other four domains. Overall quality was also assessed at one of the three levels based on the total count under each rating level. A detailed assessment of quality is presented in Table S2.

## 3. Results

By searching for relevant studies published in English and Japanese, a total of 40 eligible studies [15,16,17,18,19,20,21,32,44,45,46,47,48,49,50,51,52,53,54,55,56,57,58,59,60,61,62,63,64,65,66,67,68,69,70,71,72,73,74,75] were included in this review (Figure S1), 2 of which were published in Japanese [55,66]. Table 1 shows the characteristics of included studies and selected results; Table S3 presents more detailed information. All studies were published after the year 2000 and based on cross-sectional analyses. The median sample size was 1049 (range, 50–58,051). Most of the studies (80%, *n* = 32) were conducted in English-speaking countries: the United States (US: *n* = 22), the United Kingdom (UK: *n* = 4), Australia (*n* = 2), Canada (*n* = 2), and Ireland (*n* = 2). Eight studies were conducted in non-English-speaking countries: Japan (*n* = 5), Korea (*n* = 2), and Brazil (*n* = 1). Twenty-eight (70%) studies exclusively targeted populations aged ≥18 years, ten (25%) focused solely on those aged <18 years, and two (5%) focused on both.

Almost all (90%, *n* = 36) the studies investigated associations between HC and dietary variables, with the remaining four studies assessing the contribution of home-cooked food products to dietary intake. For dietary variables, 6 studies focused exclusively on diet quality, 27 exclusively focused on dietary intake, and the other 7 on both. Table 1 shows the frequently-assessed dietary variables in the studies included in the review, namely, the intakes of fruits and vegetables (separately or together), fast foods/snacks/sugar/SFA, and salt/sodium (Table S3 presents the detailed results).

### 3.1. Definitions of “Home Cooking”

The eight studies [15,17,44,45,46,47,48,49] that defined HC (Table 2) were classified into two main themes: level of food preparation (*n* = 7) and time spent on food preparation (*n* = 1). For studies focused on the level of food preparation, three sub-themes were further identified, namely, the exclusion of specific foods (*n* = 2), indication of ingredients (*n* = 2), and provision of examples (*n* = 3). Two studies defined the concept of “from scratch” [44,45] under the sub-themes of the exclusion of specific foods and provision of examples. One definition under the sub-theme of the indication of ingredients used the word “scratch” to define “home-cooked meals” [17]. For the remaining studies, the definitions of “food-prepared at home” [46], “food preparation” [49], and “cooked meals” [47,48] were classified under each of the subthemes. One study used a definition based on the time spent on food preparation with a cutoff value of 10 min [15].

### 3.2. Methods for Assessing “Home Cooking”

For studies using perception-dependent methods (*n* = 29), 27 used single indicators, and 2 used multiple indicators to assess HC (Table 1). Except for one study [65], none used a validated method. The perception dependent methods were based on 22 sets of questions, with 13 used the terms related to “prepare” [44,45,46,49,54,55,56,57,58,59,61,63,64] and 6 used the terms related to “cook” [15,16,48,50,62] (Table S4). For the rest, one each measured “dinner made at home” [60], “food-choice coping strategies” [65], and “ways of eating (breakfast, lunch, or dinner)” [66]. Eighteen studies used the frequency of food preparation to assess HC [15,18,19,20,44,45,47,48,49,50,51,52,53,54,55,56,57,58], whereas 7 used the frequency of consumption [33,46,59,60,61,62,63]. One study each assessed HC based on the food preparation location [55] and level of preparation [17]. Two studies identified patterns or clusters of food preparation based on multiple indicators [64,65]. In addition to the frequency of meal preparation [64,65], Martins et al. [64] also included indicators such as meal planning, time spent on cooking, and cooking skills to assess domestic food preparation practices. Three practice patterns were identified (based on principal component analysis): “healthy cooking”, “usual cooking”, and “convenience cooking”. Blake et al. [65], however, specifically targeted working parents and used indicators to reflect family meal preparation (e.g., “includes canned or frozen entrees or boxed mixes”) and individual food behaviors (e.g., “on busy days, you eat a meal in the car”). “Home cooking” was one of the three clusters identified in the study (the other two were “individualized” and “missing meals”) [65]. 

For studies using perception-independent methods (*n* = 11), 8 used single indicators, and three used multiple indicators to assess HC (Table 1). The food preparation location [67,68,69,70] was the most frequently used indicator (*n* = 4), followed by the location of food obtained or purchased (*n* = 3) [32,71,72]; one study used the level of preparation based on whether a “minimal level of preparation” was required before consumption [73] (Table S4). For those based on the indicator of location, 6 studies intended to measure “home food” or “home meal” [32,67,69,70,71,72], with one measuring “dinner eaten at home” [68]. For the rest, all aimed to measure “home-prepared foods” [21,73,75] or “at-home food preparation” [74]. McLaughlin et al. [74] and Astbury et al. [21,75] used multiple indicators for assessing HC based on contextual information obtained from a 3-day, 24-h dietary recall, and a 3- or 4-day diet record, respectively. McLaughlin et al. [74] developed and validated a regression equation to predict the “complexity of food preparation” and applied the predicted “complexity” score to identify a “meal prepared from scratch”. The response variable, “complexity of food preparation”, was defined by the total frequency of the five food preparation techniques (i.e., washing, subdivision and fraction, combining and mixing, heating, and the removal of heat) applied on each eating occasion. Potential predictors of complexity included the presence of a recipe(s), the number of foods included in the recipe(s), the number of foods not included in the recipe(s), and the time of consumption or the eating occasion. The number of foods included and not included in the recipe(s) was selected as predictors in the final equation [74].

Rather than using a mathematical method for assessing HC, Astbury et al. [21,75] identified “home-prepared food” by excluding foods that were “not home-prepared” (e.g., “foods requiring the application of heat or the addition of hot water but no other preparation”). The indicators used for classification included food preparation location (e.g., restaurant), food type (e.g., instant noodles were excluded from home-prepared dishes), recipe (i.e., whether foods were prepared with a recipe), and recipe type (i.e., whether the recipe was manufactured) [21,75]. Based on this method, foods that required limited preparation (e.g., sandwiches (because they were classified as “no recipe”)) were excluded from “home-prepared foods” [21,75].

### 3.3. Dietary Assessments

The dietary variables of 22 (55%) studies were assessed using 24-h dietary recall or diet records (Table 1). For the rest (*n* = 18), only seven (37%) assessed all dietary variables based on validated assessment methods [15,16,19,45,49,56,57,59,60], whereas remainder used at least one non-validated method for a dietary variable [17,44,47,48,57,58,60,61,63,66]. Although dinner or the main meal was the most frequent meal occasion for HC analysis, most dietary variables were not assessed based on specific meal occasions; four studies specifically analyzed dinner [50,51,64,68]. Assuming that the studies did not specify meal occasions for HC and intended to assess daily HC practices, 68% (*n* = 27) of the studies were evaluated as concurrent regarding meal occasions. For example, both HC and dietary variables were assessed at dinner [50,51,64,68].

### 3.4. Quality Assessment of Analyses and Result Consistency

Seven studies [21,32,68,69,71,72,75] were assessed as having high ratings for overall quality (Table 1 shows the results of the overall quality assessment, and Table S5 presents the detailed quality assessment). Fourteen studies had moderate overall ratings [18,19,20,23,50,52,53,56,64,65,67,70,73,74]. All studies with low overall ratings (*n* = 19) used perception-dependent methods with single indicators for HC assessment [15,16,17,44,45,46,47,48,49,54,55,57,58,59,60,61,62,63,66] (Table 1). All studies that defined HC had low overall quality. Inconsistent findings were observed for diet quality (significantly positive relationship (hereafter, “positive”), *n* = 2; null relationship (hereafter, “null”), *n* = 2; positive and null, *n* = 1). In contrast to studies with high or moderate overall ratings, more consistent findings were found for low-rated studies in terms of fruits and vegetables (positive, *n* = 11; null, *n* = 1; positive and significant negative relationships (hereafter, “negative”), *n* = 1), whereas more inconsistent findings were found for fast foods/snacks/sugar/SFA (null, *n* = 4; negative, *n* = 2; negative and null, *n* = 1; positive and null, *n* = 1). Only one study assessed sodium levels and reported a null finding.

All studies that used perception-independent methods had high [21,32,68,69,71,72,75] or moderate [67,70,73,74] overall ratings. Inconsistent findings were observed for diet quality (positive, *n* = 4; null, *n* = 3), fruits and vegetables (positive, *n* = 5; null, *n* = 3; positive, and negative, *n* = 1), and sodium (null, *n* = 2; negative, *n* = 2; positive, *n* = 1; negative and null, *n* = 1). More consistent findings were observed for fast foods/snacks/sugar/SFA (negative, *n* = 8; null, *n* = 1), for which all studies presented results at the nutrient level (e.g., SFA and sugar) [19,20,21,50,53,65,68,71], except for one that assessed the percentage of energy from ultra-processed foods [64].

## 4. Discussion

Research has suggested that HC is associated with healthy eating habits. However, as HC is a complex concept that can be perceived and assessed from various aspects, the interpretation of research findings may be biased without considering the definition and investigation method used in a study. This is the first review to identify the definitions and methods in studies investigating the relationship between HC and dietary variables. Of the 40 studies identified in this review, 8 provided definitions of HC. Most of the studies (*n* = 27) used perception-dependent methods with single indicators (e.g., frequency of food preparation) to assess HC. Few studies (*n* = 3) used perception-independent methods with multiple indicators. Nearly half (*n* = 19) of the studies were rated low for overall quality, followed by 14 moderate and 7 high-quality studies. Although more evidence is needed, our review demonstrated that the relationship between HC and dietary variables might be biased depending on the quality of analysis.

### 4.1. Definitions of “Home Cooking”

Previous quality and mixed-method studies have indicated that HC can be perceived in a range from foods prepared mostly from raw ingredients to microwaved ready-to-eat foods [25,28,29]. The perceptions of HC may vary across life stages [29] and be influenced by personal (e.g., income, perceived time pressure), sociocultural (e.g., family history, ethnicity), and environmental (e.g., food availability in the market) factors [22,23,24,25,26,29,31]. Therefore, to overcome the potential heterogeneity in the perceptions toward HC at some level, investigators may have to provide their definitions; otherwise, the results (e.g., groups divided by the frequency of preparation) may vary for participants with different perceptions regarding HC. For example, when one considers only cooking from scratch as the proper method of food preparation, usual food preparation (e.g., creating a meal from dishes that use raw and pre-prepared ingredients) may not be considered HC [25]. On the contrary, people who consider microwaving a frozen meal as an HC method may report a high frequency of HC [26,28].

Although several studies in this review defined the terms related to HC, their definitions were potentially ambiguous. Three studies targeted the idea of “scratch” [17,44,45]. Similar to the notion of HC, perceptions toward the concept of “from scratch” range from meals exclusively prepared using raw ingredients to those incorporating convenience foods at some level [30,76]. Therefore, for a study that used “scratch ingredients” to define home-cooked meals [17], some examples were necessary to indicate the types of food items that could be considered “scratch ingredients”. One study defined it by excluding “box or pre-prepared mixes and sauces” [44]. Although some meal types (e.g., those prepared from pancake mixes) may be excluded from one’s perception of HC, some ambiguity may remain for food items such as sliced bread, soup stock, and pre-cut vegetables. For the study that used lasagna preparation as an example of “meal preparation from basic ingredients” [45], the authors did not specify whether tomato sauce and noodles could be used as pre-prepared ingredients.

Similar interpretations can be applied to definitions, including other terms. One study defined “food-prepared at home” as that which “has not been pre-prepared/take out/fast food” [46]. However, although a fully pre-prepared meal can be excluded by the definition, as most people prepare meals by mixing raw ingredients and convenience foods [77], researchers may need to further indicate the extent to which pre-prepared food items can be allowed in a meal. Rather than defining HC by excluding specific foods, two groups of authors showed food or meal types that could be considered HC [47,48,49]. One group referred to a “simple meal, such as fried eggs”, as a “cooked meal” [47,48]; the other indicated that “combining any two ingredients (such as cereal and milk)” could be considered “food preparation” [49]. The prevalence distribution may be skewed toward the “high” frequency group [47,48] because it could be comprised of participants who cooked more often with raw ingredients and those who frequently used microwaved ready-made meals. Therefore, the dietary variables of HC may be challenging to assess because the definitions used in these studies are not sufficiently sensitive to distinguish between populations engaging in different types of cooking (e.g., meal preparation using more raw ingredients versus more convenience foods).

One study defined HC based on the time spent on cooking with a cut-off of 10 min [15]. Despite the absence of a standard for the time spent on HC, one large-scale nationwide study in the UK reported that over 60% of women spent more than 30 min continuously cooking [78]. Moreover, a UK study also reported that cooking dinners for longer than 20 min was related to higher intakes of fiber, vegetables, meat, iron, and sodium [79]. In addition, a total daily cooking time longer than one or two hours has been reported to have a higher intake of fruits and vegetables as well in the US [80]. A cutoff time of 10 min may be too short to distinguish the differences in cooking types (e.g., people using more raw ingredients with varied techniques for preparing dinner versus people incorporating more convenience foods that only require microwaving for dinner preparation). The mix of different types of participants may partially explain the null findings reported in the study by Saito et al. [15].

### 4.2. Methods for Assessing “Home Cooking”

Terms related to “preparation” were the most frequently assessed contents for HC. Previous studies have suggested that the word “cooking” may be perceived with or without a heating procedure [23,25], and “preparation” may thus be more accurate [75]. However, procedures performed on convenient food products may therefore be perceived as home food preparation. More studies are needed to distinguish whether the use of “cooking” and “preparation” in perception-dependent methods may impact the relationship with dietary variables.

Most of the methods examined in this review used single indicator (e.g., the frequency of food preparation) based on perception-dependent methods using questionnaire [15,16,45,47,48,55,57,58,59,63,66] or interviews [19,44,49,53,54,56,64,65]. Using single indicators may not capture the complexity of HC [24]. For example, the frequency of dinner preparation was not related to fruit and vegetable intake for a low-income U.S. population [20]. However, a study conducted among low-income Canadian women using the perception-independent method with multiple indicators showed that when considering factors such as food preparation techniques and the number of foods presented at an eating occasion, the complexity of food preparation and frequency of cooking from scratch was positively related to the fruits and vegetable intake [74]. Notwithstanding the 2 studies conducted for different populations, investigating food preparation in greater detail may provide a different result. Moreover, self-reported scales are prone to reporting bias. In the case of HC, the reason for the high frequency of food preparation may stem from a social desirability bias; as reported by previous studies, HC is often viewed as “proper” behavior [25,29,31,81].

Single indicators based on perception-independent methods may also be prone to misclassification bias. For this type of study, home-cooked/prepared foods were identified based on the location (i.e., of food obtained/purchased or food preparation) information recorded in a 24-h dietary recall or diet record [32,67,68,69,70,71,72]. For the location of food obtained/purchased, although retail stores offer participants more control over food choices, convenience foods are widely available in the market [4]. Thus, home-cooked/prepared food identified by the location of obtaining food may include a mix of raw ingredients and ready-made meals. For preparation location, participants may have different perceptions regarding “preparation” (e.g., the location where ingredients are assembled versus the location of heating before serving). Therefore, the location of preparation may be difficult to identify for foods assembled and heated at different places.

As previous studies have pointed out, HC is a complex behavior related to various factors (e.g., ingredients, techniques, utensils, time spent on food preparation, or level of processing), which may interact with each other [25,33,82,83]. Using multiple indicators to assess HC may thus provide a more insightful picture. However, few attempts have been made to address this issue using multiple indicators to assess HC [64,65]. Martins et al. [64] and Blake et al. [65] assessed behavioral patterns between indicators using principal component and cluster analyses, respectively. Although Blake et al. [65] used a validated scale, the indicators were explicitly designed to assess food choice-related behaviors (e.g., food choice behaviors on workdays) among working parents. Thus, the scale may have limited utility for other populations. Moreover, Martins et al. [64] used more general indicators (e.g., time spent on cooking, cooking techniques, and meal planning) to assess HC. Still, they did not indicate the period (e.g., at a particular meal or during the past week) of the scale in question, the choice of indicators, development procedures, or validity.

Based on detailed dietary assessment methods (i.e., 24-h dietary recall or diet records), McLaughlin et al. [74] and Astbury et al. [21,75] also used multiple indicators to assess HC. McLaughlin et al. [74] used a regression method that allows investigators to choose response variables and potential predictors based on the study’s purpose and variables available in the diet records; the predictors that best explain the variation in the response vary according to the data can then be chosen during data analysis. In addition, neither the investigators nor the participants are required to classify the foods that are difficult to assign to a category (e.g., instant noodles heated at home), bypassing the variation because of diverse perceptions [76]. Moreover, the regression equation developed for a subgroup drawn from a larger group of participants, once validated (usually for another subgroup), can be applied to all participants, which is an advantage of developing regression methods for studies with a large sample size. However, the equations developed using regression methods are data driven, so they may not readily apply to other populations without further adaptation or re-development. Although assessing HC based on detailed dietary assessment methods is considered perception-independent, classification accuracy heavily depends on the availability and quality of the information obtained in the dietary assessment method. To ensure quality, investigators (and perhaps, participants) must be rigorously trained to record, check, and enter the data. Moreover, investigators must have deep knowledge and experience in handling dietary data to develop and execute the protocol. Although high-quality data and carefully designed protocols may enhance accuracy and reduce bias from subjective perceptions, the drawbacks mentioned above may limit the utility of detailed dietary assessment methods in resource (e.g., labor, funding, and time)-constrained conditions.

### 4.3. Quality Assessment of Analyses and Consistency of Dietary Variables

The results of studies on the association between HC and dietary variables were not consistent across the levels of overall quality of analysis. This could be partially explained by the heterogeneity of the methods used to assess HC and dietary variables. The relationship between HC and fruit and vegetable intake was more consistent in studies with low overall ratings, which may be partially explained by the correlation between the misreporting of methods used for assessing HC and dietary variables. All studies with low overall ratings used self-reported methods based on single indicators to assess HC. Additionally, all but two studies [16,55] used short questionnaires [44,45,57,60,62] or single questions [17,47,48,58,61,63] for assessing dietary variables. Previous studies have suggested that using short questionnaires (<16 food items) to estimate [84] or rank [84,85] fruits and vegetable intake is of limited validity. Moreover, as both HC and fruits and vegetable intake tend to be regarded as “proper” or “healthy” behaviors, the results of HC (e.g., frequency of preparation) and fruits and vegetable intake are likely to be overestimated because of the social desirability bias [81].

The inconsistent findings for fast foods/snacks/SFA/sugar between studies with low, high, or moderate overall ratings may be attributed to differences in dietary variables (i.e., at the nutrient or food level) and assessment methods. For studies with high or moderate overall ratings, except for one that used a validated food frequency questionnaire, all used 24-h dietary recall or diet records to assess SFA/sugar/sodium or all these items at the nutrient level. However, six out of eight studies with low overall ratings used either non-validated questionnaires or single questions to assess the frequency of servings of the intake of various food items (e.g., “fast foods”, “chips, candy, and pastries”) based on different reference criteria (from 1/week [58] to 2.5 times/day [44]). Therefore, between-study comparisons among studies with low ratings could have been hampered.

Except for one study, all studies analyzed diet quality based on 24-h dietary recalls, dietary records, or validated questionnaires. The inconsistency in research findings may be because of the different indicators of diet quality used. For example, the Diet Approaches to Stop Hypertension score used in the studies identified in this review was based on the relative scoring system according to the distribution of the target population [86]. In contrast, the Healthy Eating Index was scored based on a pre-determined scale [87] or incomplete confounding controls.

### 4.4. Implications for Future Research

As HC may incorporate a mix of raw ingredients and convenience foods [76], it can be challenging to assess whether some food items (e.g., sliced bread, soup stock, and pre-cut vegetables) should be classified as “scratch” or “convenience” ingredients [77]. Future studies may need to clarify the extent to which convenience foods can be allowed in HC and the kinds of convenience foods that are permitted in the study (e.g., pre-prepared dishes or ingredients). Future study-specific definitions may be generated by combining the three subthemes (i.e., exclusion of specific foods, an indication of ingredients, and providing examples) identified in this review. In addition, using the time spent on cooking as an indicator may be an alternative parameter, as its measurement is relatively objective (i.e., using a clock). The cutoff value should be determined based on research findings, although the problem of misclassification because of different food preparation practices remains. However, regardless of the main definitional themes, further examination is needed to assess whether adding definitions to the survey item on HC or specifying the definitions can make a difference in classifying participants by the different types of food preparation. It is also noteworthy that study-based definitions can be arbitrary with limited comparability across studies.

To address the issues in generating a clear definition of HC, more studies that use multiple indicators are necessary to reflect the complexities of HC. When the objective is to investigate HC as a behavior (e.g., a food preparation practice), future studies may use a self-reported questionnaire by including multiple indicators related to cooking practices (e.g., the frequency of preparation, time spent on food preparation, ingredients included in food preparation, and cooking skills) selected based on available evidence and further tested for validity and reliability [88,89,90]. Self-reported scales are relatively inexpensive and easy to administer, whereas direct observation of cooking behavior can be labor-intensive and time-consuming, which is not feasible in a study with large participants. 

To study the consumption of home-cooked food products, it may be suggestive to use the perception-independent method as it is not dependent on participants’ interpretations and better reflects the study objective. Studies have also suggested incorporating home-prepared foods into a wider extent of food processing classification while considering the ingredients used in the food preparation [76,83]. Other than the methods extracted in this review [21,74,75], a recent study [83] also proposed a food processing framework to include classifications of home-prepared foods, and processed food classification for categorizing home-prepared foods, by incorporating information on the processing of the component ingredients. For example, if home food preparation involved highly-processed culinary ingredients (e.g., tomato sauce) or moderately processed foods (e.g., smoked or cured meat), then the food would be classified as moderately processed food; if the food only contained basic or unprocessed foods (e.g., plain milk and whole grain pasta), then the food would be classified as basic processed home-prepared food. This framework may be useful in categorizing home-prepared foods as distinct from industrially produced foods. Contextual information collected based on detailed assessment methods may be valuable in assisting investigators in classifying home-prepared food products [91]. For example, meal occasions may be recorded according to their components (e.g., dishes, foods, and beverages) along with their preparation locations. Other information, such as the time spent on preparation and the techniques and utensils used for preparation, may also be included. However, recording additional information may burden participants and investigators, further limiting its feasibility. Future studies may thus consider utilizing portable devices with validated applications to study HC and dietary intake for capturing “real-time” behaviors [17,92].

### 4.5. Limitations

First, although this review utilized several databases, it may not have covered all the relevant studies owing to the presence of unexplored databases and the restriction of the sample to studies published in English and Japanese. Second, the definitions and assessment methods of HC identified in this review may not apply to contexts other than those of the developed Western countries (e.g., East Asian developing countries, Middle East countries). Although seven of the studies included in this review were based on data collected in East Asia (i.e., Japan and Korea), the definitions (e.g., simple meal, cooking dinner in <10 min) and assessment methods (e.g., food preparation location) may not reflect the expected differences in cooking cultures (e.g., meals consisting of multiple dishes with varied ingredients [93]). Third, the criteria used to assess the quality of the included studies were adapted to fit the purpose of this review; as a result, they may not be applicable for assessing the overall quality of the studies.

## 5. Conclusions

This review identified the definitions and methods used to assess HC. Although a consensual definition of HC is challenging to generate, study-specific definitions may be necessary to clarify the boundaries between the foods or behaviors deemed HC and those not, based on the study’s objective. The definitions extracted in this review did not describe the level and extent to which convenience foods should be included in HC. Future studies should focus on generating definitions with greater clarity.

Although HC is a complex concept, most of the sampled studies assessed it using methods that depended on participants’ perceptions and were based on single indicators. HC assessments based on perception-independent methods may be more objective as they do not rely on participants’ perceptions. However, detailed dietary assessment methods (e.g., 24-h dietary recalls) are necessary for applying such methods, which may not always be feasible. An alternative could be a self-reported questionnaire with multiple indicators. In addition, incorporating portable devices into research may be helpful in capturing real-time data while reducing the burden on the participants and investigators. The quality of analysis may explain the inconsistent findings regarding the relationship between HC and dietary variables. Although HC has been reported to be related to an increased fruit and vegetable intake, the review findings showed that the results might be biased because of poor quality of analyses using single indicators and perception-based methods, along with non-validated dietary assessment methods. To better understand the relationship between HC and dietary variables, future studies should clarify the definition of HC and assess it with various indicators.

## Figures and Tables

**Table 1:** Characteristics and selected results of the included studies (*n* = 40).

							Results of Selected Dietary Variables ^4^				
First Author, Year of Publication	Country	Terms Regarded as HC	Definition Provided (Yes/No) ^1^	Perception-Dependent/-Independent ^2^	Indicator	Dietary Assessment; Validation (Yes/No/NA) ^3^	Diet Quality ^5^	Intake of Fruits and Vegetables ^6^	Intake of Fast-Foods/Snacks/Sugar/SFA ^7^	Intake of Salt/Na	Overall Quality of Analyses
Fertig A, 2019 [17]	US	Home-cooked meals	Yes	Dependent	Level of preparation	Mealtime ecological momentary assessment ^8^, 8 days; no		↑			Low
Gustat J, 2017 [44]	US	Preparation from scratch	Yes	Dependent	Frequency (preparation)	Questions for target dietary items; no		↑	↔		Low
Hanson A, 2019 [45]	US	Meal preparation from basic ingredients	Yes	Dependent	Frequency (preparation)	Screener (past month’s fruit and vegetable intake); yes		↑			Low
Pachucki M, 2018 [46]	US	Food-prepared at home	Yes	Dependent	Frequency (consumption)	24 h DR, 2 days; (NA)	↑/↔				Low
Saito A, 2019 [15]	Japan	Cook dinner at home	Yes	Dependent	Frequency (preparation)	BDHQ (past month, 58 items); yes	↔				Low
Sattler M, 2015 [49]	US	Food preparation	Yes	Dependent	Frequency (preparation)	FFQ (77 items); yes	↔		↔	↔	Low
Tani Y, 2020 [47]	Japan	Cooked meals at home	Yes	Dependent	Frequency (preparation)	Single question for target dietary items; no		↑			Low
Tani Y, 2019 [48]	Japan	Cooked meals at home	Yes	Dependent	Frequency (preparation)	Single question for target dietary items; no		↑			Low
Farmer N, 2019 [50]	US	Cook food for dinner	No	Dependent	Frequency (preparation)	24 h DR, 1 day; (NA)	↔	↑	↓	↔	Moderate
Farmer N, 2020 [51]	US	Cook food for dinner	No	Dependent	Frequency (preparation)	24 h DR, 2 days; (NA)		↑		↑	Moderate
Wolfson J, 2020 [18]	US	Cook food for dinner	No	Dependent	Frequency (preparation)	24 h DR, 2 days; (NA)	↑				Moderate
Wolfson J, 2015a [52]	US	Cook food for dinner	No	Dependent	Frequency (preparation)	24 h DR, 1 day; (NA)		↑/↓			Moderate
Wolfson J, 2015b [53]	US	Cook food for dinner	No	Dependent	Frequency (preparation)	24 h DR, 1 day; (NA)			↓		Moderate
Taillie L, 2017 [20]	US	Cook food for dinner	No	Dependent	Frequency (preparation)	24 h DR, 1 day; (NA)		↔	↓		Moderate
Tiwari A, 2017 [19]	US	Cook food for dinner	No	Dependent	Frequency (preparation)	FFQ (previous year, 125 items); yes	↑	↑	↓	↔	Moderate
Lam M, 2017 [54]	UK	Prepare main meal	No	Dependent	Frequency (preparation)	Diet record, 4 days; (NA)			↓		Low
Ozawa K, 2018 (in Japanese) [55]	Japan	Prepare meals	No	Dependent	Frequency (preparation)	Diet record, 2 days; (NA)		↑			Low
McGowan L, 2016 [56]	Ireland	Prepare meals aside from main meal	No	Dependent	Frequency (preparation)	Short questionnaire for each diet quality and dietary intake; yes	↑				Moderate
Laska M, 2015 [57]	US	Prepare meal at home; prepare dinner	No	Dependent	Frequency (preparation)	Short questionnaire; yes/no ^9^		↑/↓	↔		Low
Bassul C, 2020 [58]	Ireland	Prepare meals at home	No	Dependent	Frequency (preparation)	Single question for target dietary items; no		↔	↔		Low
Mills S, 2017 [16]	UK	Home cooked meals	No	Dependent	Frequency (consumption)	FFQ (previous year, 130 items); yes	↑	↑			Low
Zong G, 2016 [59]	US	(Midday or evening) meals prepared at home	No	Dependent	Frequency (consumption)	FFQ (previous year, 138 items); yes	No statistical test conducted				Low
Appelhans B, 2014 [60]	US	Dinner made at home	No	Dependent	Frequency (consumption)	Short questionnaire; yes/no		↑	↓		Low
Erinosho T, 2012 [61]	US	Meals prepared at home	No	Dependent	Frequency (consumption)	Questions for target dietary items; no		↑	↓/↔		Low
Overcash F, 2020 [62]	US	Food cooked from scratch or a recipe	No	Dependent	Frequency (consumption)	Short questionnaire (past 7 days, 27 items); no		↑	↑/↔		Low
Crawford D, 2006 [63]	Australia	Meals prepared at home	No	Dependent	Frequency (consumption)	Questions for target dietary items; no		↑			Low
Martins C, 2021 [64]	Brazil	Food preparation practice	No	Dependent	Frequency (preparation); time of preparation; meal planning; food skills; cooking skills; confidence in cooking	24 h DR, 2 days; (NA)			↓		Moderate
Blake C, 2011 [65]	US	Food-choice coping strategies	No	Dependent	Frequency (consumption); ready meal use; meal planning	24 h DR, 2 days; (NA)	↔	↔/↑ ^1^^0^	↔		Moderate
Yoshiba K, 2015 (in Japanese) [66]	Japan	Ways of eating (breakfast, lunch, or dinner)	No	Dependent	Location of food preparation	Dietary diversity: qualitative questionnaire ^11^; no	↑				Low
Kwon Y, 2018 [67]	Korea	Home meal	(NA)	Independent	Location (preparation)	24 h DR, 1 day; (NA)		↔			Moderate
Kim S, 2018 [68]	Korea	Dinner eaten at home	(NA)	Independent	Location (preparation)	24 h DR, 1 day; (NA)			↓	↓	High
Nishi S, 2018 [69]	Canada	Home food	(NA)	Independent	Location (preparation)	24 h DR, 1 day; (NA)	Studied the proportion of home-cooked food products in individual dietary intakes				High
Wellard-Cole L, 2021 [70]	Australia	Home food	(NA)	Independent	Location (preparation)	Diet record (smartphone application), 3 days; yes	Studied the proportion of home-cooked food products in individual dietary intakes				Moderate
Guthrie J, 2002 [71]	US	Home food	(NA)	Independent	Location (obtained/purchased food)	24 h DR, 1 day; (NA)			↓	↓/↔	High
Smith L, 2013 [32]	US	Home food	(NA)	Independent	Location (obtained/purchased food)	24 h DR, 1 day; (NA)	Studied the proportion of home-cooked food products in individual dietary intakes				High
Smith T, 2019 [72]	US	Home food	(NA)	Independent	Location (obtained/purchased food)	24 h DR, 2 days; (NA)	Studied the proportion of home-cooked food products in individual dietary intakes				High
Appelhans B, 2012 [73]	US	Home-prepared foods	(NA)	Independent	Level of preparation	Diet record, 7 days; (NA)	Assessed for per food item energy intake and per food item energy density				Moderate
McLaughlin C, 2003 [74]	Canada	At-home food preparation	(NA)	Independent	Food preparation techniques; location (preparation); presence of recipe; number of foods per recipe; level (preparation); time the eating occasion	24 h DR, 3 days; (NA)		↑			Moderate
Astbury C, 2019a [21]	UK	Home-prepared foods	(NA)	Independent	Location (preparation); food type; recipe; recipe type	Diet record, 3 or 4 days; (NA)	↑				High
Astbury C, 2019b [75]	UK	Home-prepared foods	(NA)	Independent	Location (preparation); food type; recipe; recipe type	Diet record, 3 or 4 days; (NA)	↔	↔	↓	↓	High

^1^ Definition only extracted from the perception-dependent methods. ^2^ Methods were classified into two types, “perception-dependent” and “perception-independent”, based on if the classification of “home cooking” was dependent on participants’ perception. ^3^ “NA” for 24 h DR or diet record as validation study is usually not needed. ^4^ If not otherwise indicated, “↑”, “↓”, and “↔” indicate “significant positive relationship”, “significant negative relationship”, and “null relationship”, respectively, corresponding to an increased magnitude of the indicator of “home cooking”. ^5^ Diet quality included Healthy Eating Index-2005, -2010, and -2015; Diet Approaches to Stop Hypertension; Mediterranean Diet Score; Eating Choice Index; and a dietary diversity index. ^6^ Included studies that investigated fruits and vegetables separately or together. ^7^ Also included empty calories/SoFAAS (i.e., solid fat, alcoholic beverages, and added sugars)/SoFAS (i.e., solid fat and added sugars); ultra-processed foods; chips, candy, and pastries; confectionary/desserts; French fries; and junk food. ^8^ By asking participants whether certain ingredients (i.e., fruits, vegetables, and whole grains) were served at the meal and whether children ate any of the served ingredients. ^9^ Validated methods used for assessing intakes of fast food, fruits, and vegetables, but not for sugar-sweetened beverages. ^1^^0^ “↑” for dark green vegetables and “↔” for total vegetables. ^11^ Weekly consumption (as binary, namely, “everyday” and “not everyday”) for 10 food groups (i.e., meats, fish, eggs, dairy, legumes, dark green/orange vegetables, seaweed, fruits, tubers, and fat/oils) with a total score of 10. DR, dietary recall; FFQ, food frequency questionnaire; BDHQ, brief-type self-administered diet history questionnaire; HC, home cooking; NA, not applicable; SFA, saturated fatty acid.

**Table 2:** Definitions of “home cooking” are indicated in the included studies.

Main Themes	Sub-Themes	Definitions	First Author, Year of Publication
Level of food preparation	Exclusion of specific foods	“‘scratch’ refers to meals prepared at home without box or pre-prepared mixes and sauces.”	Gustat J, 2017 [44]
		“... meals per week do you eat that have been prepared at home (meaning that food that has put together and cooked yourself (or by someone else in the household) and has not been pre-prepared/take out/fast food).”	Pachucki M, 2018 [46]
	Indication of ingredients	“..., this study defines a fully home-cooked meal as one made at home from mostly scratch ingredients”; “partly home-cooked meals are those made from a combination of scratch ingredients, restaurant food and/or pre-prepared foods.”	Fertig A, 2019 [17]
		“Food preparation included combining any two ingredients (such as cereal and milk), or the heating of a food item (such as baking frozen chicken nuggets).”	Sattler M, 2015 [49]
	Provision of examples	“Cooking frequency was assessed using one question asking how often per week participants prepared meals from basic ingredients such as combining ground beef, tomato sauce, cheese, and noodles to make lasagna.”	Hanson A, 2019 [45]
		“A cooked meal is defined as a simple meal, such as fried eggs.”	Tani Y, 2020 [47]; Tani Y, 2019 [48]
Time spent on food preparation	(NA)	“..., how many times per week did you cook dinner at home for your family and for yourself? This includes working in the kitchen for more than 10 min, and also includes helping someone to cook. Do not include preparing tables or washing dishes.”	Saito A, 2019 [15]

## Data Availability

Not applicable.

## Supplementary Materials

The following supporting information can be downloaded at: https://www.mdpi.com/article/10.3390/nu14163344/s1, Figure S1: Flow diagram of selection of studies; File S1: Search strategies over databases; Table S1: Eligibility criteria for study selection; Table S2: Quality assessment of analyses; Table S3: Additional information of the included studies (*n* = 40); Table S4: Methods of assessing “home cooking”; Table S5: Quality assessment of the included studies.
