# Supplementary material for: Definitions and Assessment Methods of ‘Home Cooking’ in Studies with Dietary Variables: A Scoping Review
Source: Nutrients. 2022 Aug 15;14(16):3344. doi: 10.3390/nu14163344 (PMC9412361; doi:10.3390/nu14163344)
Supplement: Supplementary file 1 [file nutrients-14-03344-s001.zip › nutrients-1815808-supplementary-v2.pdf]

Supplementary files

Definitions and assessment methods of “home cooking” in studies evaluating its relationships with dietary variables: A scoping review

Figure S1. Flow diagram of selection of studies.

File S1. Search strategies over databases

Table S1. Eligibility criteria for studies selection.

Table S2. Quality assessment of analyses.

Table S3. Additional information of the included studies (n=40).

Table S4. Methods for assessing "home cooking".

Table S5. Quality assessment of the included studies.

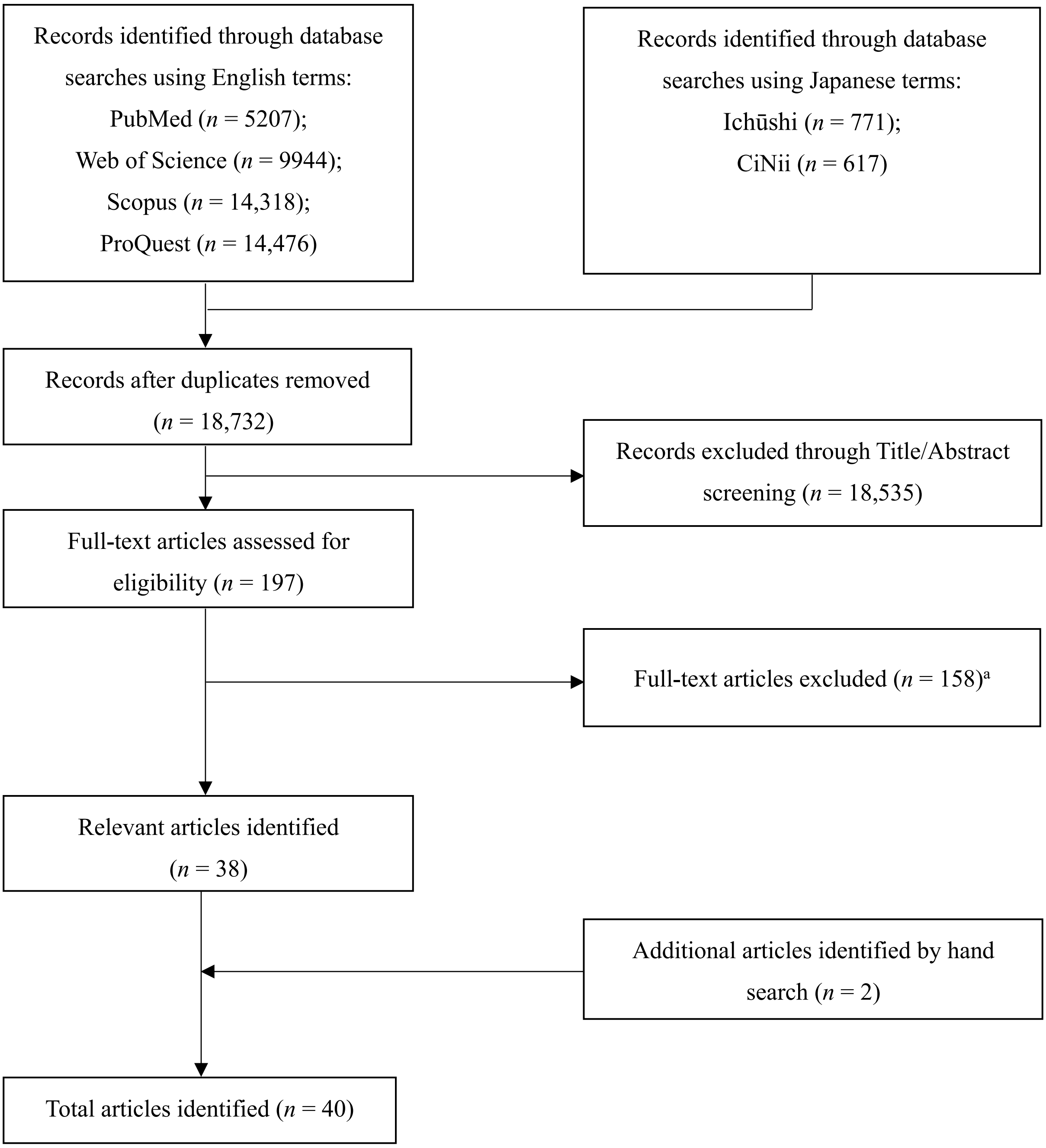

Figure S1. Flow diagram of selection of studies. [a] No full text accessible (*n*=3); not target study design (*n* =10); not targe participants (*n* =0); no measurement/ direct measurement on home cooking (*n* =104); foods for home cooking were exclusively on specific items (e.g., home-cooked meat, vegetables prepared at home) (*n* =1); no dietary variables (*n* =29); no association or contribution of nutrient intake has been studied (*n* =12).

File S1. Search strategies over databases

1. **PubMed** (Last search: June 14, 2021. *N*=5,207. Filter: English, Japanese)

4	#1 and #2 and #3
3	cooking[Title/Abstract] OR cook[Title/Abstract] OR cooks[Title/Abstract] OR cooked[Title/Abstract] OR prepar*[Title/Abstract] OR make[Title/Abstract] OR made[Title/Abstract] OR making[Title/Abstract] OR makes[Title/Abstract]
2	food[Title/Abstract] OR foods[Title/Abstract] OR meal[Title/Abstract] OR meals[Title/Abstract] OR dinner*[Title/Abstract] OR supper*[Title/Abstract] OR breakfast*[Title/Abstract] OR lunch*[Title/Abstract] OR diet[Title/Abstract] OR diets[Title/Abstract] OR dish[Title/Abstract] OR dishes[Title/Abstract] OR dietary[Title/Abstract] OR eating[Title/Abstract] OR eat[Title/Abstract] OR eats[Title/Abstract] OR ate[Title/Abstract] OR intake*[Title/Abstract] OR cuisine*[Title/Abstract] OR culinary[Title/Abstract] or menu*[Title/Abstract] OR recipe*[Title/Abstract]
1	home[Title/Abstract] OR homely[Title/Abstract] OR from-home[Title/Abstract] OR at-home[Title/Abstract] OR domestic[Title/Abstract] OR household*[Title/Abstract] OR homemade[Title/Abstract]

2. **Web of Science** (Last search: June 13, 2021. *N*=9,944. Indexes: SCI-EXPANDED, SSCI, A&HCI, CPCI-S, CPCI-SSH, BKCI-S, BKCI-SSH, ESCI, CCR-EXPANDED, IC. Timespan: All years)

# 4	(#3) *AND* LANGUAGE: (English) *AND* DOCUMENT TYPES: (Article)
# 3	(#1 or #2) *AND* DOCUMENT TYPES: (Article)
# 2	(AB=((cooking or cook or cooks or cooked or prepar* or make or made or making or makes) and (food or foods or meal or meals or dinner* or supper* or breakfast* or lunch* or diet or diets or dish or dishes or dietary or eating or eat or eats or ate or intake* or cuisine* or culinary or menu or recipe*) and (home or homely or from-home or at-home or domestic or household* or homemade))) *AND* DOCUMENT TYPES: (Article)
# 1	(TI=((cooking or cook or cooks or cooked or prepar* or make or made or making or makes) and (food or foods or meal or meals or dinner* or supper* or breakfast* or lunch* or diet or diets or dish or dishes or dietary or eating or eat or eats or ate or intake* or cuisine* or culinary or menu or recipe*) and (home or homely or from-home or at-home or domestic or household* or homemade))) *AND* DOCUMENT TYPES: (Article)

3. **Scopus** (Last search: June 27, 2021. *N*=14,318)

(TITLE-ABS-KEY ((cooking OR cook OR cooks OR cooked OR prepar* OR make OR making OR made OR makes)) AND TITLE-ABS-KEY ((food OR foods OR meal OR meals OR dinner* OR supper* OR breakfast* OR lunch* OR die OR diets OR dish OR dishes OR dietary OR eating OR eat OR eats OR ate OR intake* OR cuisine* OR culinary OR menu OR recipe*)) AND TITLE-ABS-KEY ((home OR homely OR from-home OR at-home OR domestic OR household* OR homemade))) AND (LIMIT-TO (PUBSTAGE , "final") OR LIMIT-TO (PUBSTAGE , "aip")) AND (LIMIT-TO (DOCTYPE , "ar")) AND (LIMIT-TO (LANGUAGE , "English") OR LIMIT-TO (LANGUAGE , "Japanese"))

4. **ProQuest** (Last search: July 11, 2021. *N*=14,476)

((((ti((cooking OR cook OR cooks OR cooked OR prepar* OR make OR made OR making OR makes) AND (food OR foods OR meal OR meals OR dinner* OR supper* OR breakfast* OR lunch* OR diet OR diets OR dish OR dishes OR dietary OR eating OR eat OR eats OR ate OR intake* OR cuisine* OR culinary OR menu OR recipe*) AND (home OR homely OR from-home OR at-home OR domestic OR household* OR homemade))) OR (ab((cooking OR cook OR cooks OR cooked OR prepar* OR make OR made OR making OR makes) AND (food OR foods OR meal OR meals OR dinner* OR supper* OR breakfast* OR lunch* OR diet OR diets OR dish OR dishes OR dietary OR eating OR eat OR eats OR ate OR intake* OR cuisine* OR culinary OR menu OR recipe*) AND (home OR homely OR from-home OR at-home OR domestic OR household* OR homemade))) AND stype.exact("Scholarly Journals")) AND la.exact("English" OR "Japanese")) AND PEER(yes))

5. Ichūshi (Last search: January 31, 2022. *N*=771)[1]
((家/AL or 宅/AL) and ((料理/AL or 料理/TH) or (調理/AL or 調理/TH)) and 食/AL) and (PT=原著論文)

6. CiNii (Last search: June 13, 2021. *N*=617)[1]
(家 OR 宅) & (料理 OR 調理) & 食

[1] Terms related to "home" (i.e., *ie* (家) or *taku* (宅)), "cooking" (i.e., *ryōri* (料理) or *chōri* (調理)), and "eating" (or "food") (i.e., *shoku* (食) because the same Chinese character is shared by *taberu* (食べる) [to eat] and *shokuhin* (食品) [food])

Table S1. Eligibility criteria for studies selection.

Eligible	Ineligible
1. Original epidemiologic studies, excluding reviews, qualitative study, study protocol or case study, that targeted on human beings<br>2. Studies aimed to examine the behaviors of food preparation practices (not assistance) and/or food consumption.<br>3. Regardless of age, health conditions, pregnancy, or whether on special dietary habits (e.g., a vegetarian diet), studies with the target participants that met the following criteria were included:<br>- People who were able to live independently and consume a normal diet<br>- People who were living in an environment where they could cook freely during the time the study was conducted<br>4. Studies that met any of the following criterion:<br>- Investigated the association between home cooking[a] and dietary variables[b]. Home cooking has to be assessed on daily or meal (e.g., dinner) basis but not on food/ingredient (e.g., home-cooked vegetables/meat, salt added during cooking) basis<br>- Analyzed the contribution of home-cooked food products to energy, nutrients, or food intake on daily or meal basis, rather than to specific food/dish.	1. No target study design (e.g., not original study, not epidemiologic study, no human beings participants, etc.)<br>2. No target participants. Namely, people who were unable to live independently or needed to consume specially processed foods (e.g., infant foods, tube feedings, etc.), or those who were living in an environment where they could not cook freely at the time when primary studies conducted (e.g., hospitals, dormitories without kitchen facilities, etc.)<br>3. No measurement or direct measurement on home cooking. Examples are as follows:<br>- Exclusively on take-away food/meal or eating outside home<br>- Family meal or eating together regardless of location of food preparation<br>- Exclusively focused on specific indicators of cooking (e.g., healthy cooking, cooking skills/methods/utensils, etc.) or factors that lead to cooking (e.g., confidence in cooking)<br>- Exclusively on meal preparation assistance<br>4. Focused exclusively on specific foods for home cooking (e.g., home-cooked vegetables/meats, salt added during cooking)<br>5. No dietary variables[b]. For examples, studies used food purchasing rather than intake assessment, based on population rather than individual or household data, or targeted on chemical or toxic substances<br>6. No association investigated between home cooking and dietary variables, or no contribution from home-cooked products to dietary intake analyzed

[a] The term home cooking also used to refer to terms having similar meanings, such as cooking at home, food/meal cooked at home, home food preparation, food/meal prepared at home, or cooking from scratch (basic ingredients) at home, but not to refer certain aspects of home cooking (e.g., cooking skills, time spent on cooking, and meal planning), or factors leading to the practice of home cooking (e.g., confidence or attitudes in cooking).

[b] Dietary variables include energy, nutrients or food intake, diet quality [e.g., *a priori* patterns using scores or index such as Healthy Eating Index 2015 (HEI-2015) and Dietary Approaches to Stop Hypertension (DASH), adherence to dietary guidelines, or *a posteriori* dietary pattern extracted by statistical methods such as principal component analysis].

Table S2. Quality assessment of analyses.

Components	High	Moderate	Low
1.Representative-ness[a]	• Used national representative dataset (e.g., US National Health and Nutrition Examination Survey, UK National Diet and Nutrition Survey), or<br>• Not exclusively recruited by using convenient sampling method and with participation rate ≥80%	• Not exclusively recruited by using convenient sampling method and with participation rate between 60 - 79%	• Used exclusively convenient sampling method, or<br>• Participation rate <60%, or<br>• No information provided
2. Confounding[b]	Controlled for at least 80% of confounders	Controlled for 60 - 79% of confounders	Controlled <60%, or not controlled or not stated
3. Methods of 'home cooking'	Used multiple indicators regardless of whether the method is perception-independent or -dependent	Used single indicator but perception-independent	Used single indicator but perception-dependent
4. Methods of dietary variables	Used 24-hour dietary recall, or dietary record	Using validated food frequency questionnaire or screener	Used unvalidated dietary assessment methods
5. Concurrence of meal occasion[c]	Yes	No	(NA)
6. Data analysis[d]	Multivariate analysis or adjustment to be national representative	Univariate analysis	(NA)
**Overall quality**	• 5 - 6 domains rated as "high" with no "low" rating" (if all 6 domains assessed), or<br>• 4 - 5 domains rated as "high" with no "low" rating (if 5 domains assessed)	• 1 "low" rating or <5 "high" ratings (if all 6 domains assessed), or<br>• 1 "low" rating or <4 "high" ratings (if 5 domains assessed)	• ≥2 "low" ratings or 1 "low" rating with ≥3 "moderate" ratings

NA, not applicable.

[a] No rating for studies referred the items to previous design study or other studies.

[b] Potential confounding factors are age (oneself/parent), gender/sex, ethnicity/race, education (oneself/ parent), income (oneself/household), employment (oneself/parent), household structure (including number of household members, number of children, or marital status). Consideration of confounding can be addressed by randomization, restriction, matching, stratification, or multivariable methods. No rating for studies that assessed trend in a population instead of analysis at individual level.

[c] Assessed at two levels: "strong" and "moderate".

[d] Assessed at two levels: "strong" and "moderate". "NA" for those studied contributions of HC food products to dietary intakes.

Table S3. Additional information of the included studies (*n*=40).

First author, year of publication	Study name, study year	Participants (age range or mean age); sample size (participation rate)[a]	Concurrence [b] (Yes/No)	Data analysis	Covariates	Dietary variables [c]	Findings related with HC [d]
Gustat J, 2017[1]	(NR), 2013	Primary household shoppers from low-income predominantly African American urban neighborhood in New Orleans, Louisiana (≥18y); n 901 (65%)	Yes	Multivariable logistic regression	Variable with p-value <0.05 at the bivariate level. Potential variables: age, gender, race/ethnicity, education, household income, BMI, food assistance program, car ownership, box mix food, fast food, distance to store patronized, shopping frequency [variables different between food groups]	Intake (frequency, daily): fresh fruits and vegetables, total fruits and vegetables; chips, candy and pastries; sweetened carbonated drinks (cut points: fruits and vegetables, ≤1 serving/day; chips, candy and pastries (no beverages), ≥2.5x/d; chips, candy and pastries, and beverages, ≥4.0x/d)	Increased "daily times of cooking from scratch (cooking at home)":<br>↑ Fresh fruits and vegetables, and total fruits and vegetables<br>↔ Chips, candy and pastry (including/excluding regular soda)
Hanson A, 2019[2]	GetFRUVED, 2015	First-year college students who consuming <2 servings of fruits or 3 servings of vegetables (≥18y), n 1108 (40%)	Yes	Multiple linear regression for determining of cooking patterns and log-transformed fruit and vegetable intake	Sex (other variables were not significantly associated with FV intake from bivariate analysis)	Intake (cup equivalent, daily): fruits and vegetables	Higher "cooking frequency":<br>↑ Fruit and vegetable intake
Fertig A, 2019[3]	Family Matters, 2015-2016	Low-income families with young children from six racial/ethnic groups (NA); n 150 households (NR)	Yes	Average predicted probabilities calculated from estimated logistic model results using within-estimator methods (identified relationship from variation within families, but not across families).	Meal type (i.e., breakfast, lunch, or snack) and day (i.e., weekday and weekend). All analyses stratified by race/ethnic groups	Intake (yes/no, daily)[c]: fruits, vegetables, whole grains	Compared with meals that described as "pre-prepared", for both "fully" and "partly" home-cooked:<br>↑ Probability of meals that children eating fruit and vegetable<br>For "fully" home-cooked only:<br>↔ Probability of children's eating whole grain intake<br>For "partly" home-cooked only:<br>↑ Probability of children's eating whole grain intake

Pachucki M, 2018[4]	Sub-study of DISTANCE, 2011-2012	Individuals with type 2 diabetes (63.3 [SD9.9] y); n 770 (57%)	Yes	Multivariable linear regression, with log-transform for energy intake because of skewness in distribution	Age, race, marital status, household income, household size, education, subjective social status, total energy; stratified by sex [based on interaction assessment]	Quality (daily): HEI-2010 and DASHIntake (energy-adjusted quantity, daily) [f]: total energy, fat, and carbohydrate	Higher frequency of "meals prepared at home": Male↓ Fat ↑ Total HEI-2010 score, and DASH adherence↔ CHO, and total energyFemale ↓ Fat↔ CHO, total energy, total HEI score, and DASH adherence
Tani Y, 2020[5]	A-CHILD, 2018	Adolescents in 2nd grade of junior high school living Adachi, Tokyo, Japan (13-14y); n 553 (87%)	Yes	Chi-square test (not main analysis)	(NA)	Intake (frequency, daily or weekly): fruit and vegetables (twice/d, once/d, <3x/wk, missing)	Higher frequency of "home cooking" [g]: ↑ Prevalence of eating twice/day of vegetable
Tani Y, 2019[6]	A-CHILD, 2018	Children from 4th grade of elementary school in Adachi City, Tokyo, Japan (9-10y); n 4258 (95%)	Yes	Chi-square test (not main analysis)	(NA)	Intake (frequency, daily or weekly): vegetable (twice/d, once/d, <3x/wk, missing)	Higher frequency of "home cooking" [g]: ↑ Prevalence of eating twice/day of vegetable
Sattler M, 2015[7]	BHCK, (NR)	Children in low-income, predominantly African American neighborhoods that are defined as food deserts (9-15y); n 289 (NR)	Yes	Multiple linear regression	Age, sex, frequency of household food preparation, and BMI percentiles	Quality (daily): HEI-2010 and its components of Na, empty calories, and dairy	Increased both frequencies of "child food preparation" and "household food preparation": ↔ Total HEI-2010, Na, empty calories, and dairy scores
Saito A, 2019[8]	POTATO, 2014-2015	Married, young-to-middle-aged women (23-44y); n 143 (68%)	No	Logistic regression for comparing the prevalence of not-meeting DRI; ANCOVA used for comparing the number of nutrients not meeting DRI	Having children or not, population size of residential area, survey year, food security, perceived diet quality (decided by univariate analysis with p<0.1) [potential variables also included education, working status, household income, current smoking status, using dietary supplement, and regions]	Quality (daily): adherence to dietary reference intakes (i.e., energy, fat, SFA, protein, CHO, dietary fiber, Na, K, vitamins A, B1, B2, B6, B12 and C, niacin, folate, Ca, Mg, Fe, Zn, and Cu)	Compared with lower frequency of "cooking dinner at home": ↔ Probability of adherences to all nutrients

Farmer N, 2019[9]	NHANES, 2007-2010	National representative Non-Hispanic Black adults (≥19y), n 2027 (NA)	Yes	Multiple linear regression	Age, gender, marital status, country of birth, employment, income to FPL, education, food security, and perceived diet quality.	Quality (daily and dinner): HEI-2010 and its components (i.e., total fruit, whole fruit, total vegetables, greens and beans, whole grains, dairy, total protein foods, seafood and plant proteins, and fatty acids, refined grains, Na, empty calories [SoFAAS])	"High" compared with "medium" and "low" frequencies of "cooking dinner at home per week":↑ Daily vegetable, daily and dinner empty calories, and dinner protein scores↓ Daily and dinner seafood and plant protein (to 2-5x/wk group only), dinner dairy scores↔ Daily and dinner total HEI scores (and scores for components other than the items listed above)
Farmer N, 2020[10]	NHANES, 2007-2010	National representative sample (≥19y), n 11481 (NA)	Yes	Dietary pattern classified by latent class profile analysis based on the 11 components of HEI-2010; multinominal logistic regression used for assessing the likelihood of socio-demographic factors being classified into a pattern	Age, sex/gender, race/ethnicity, marital status, education, employment status, poverty/income ratio, number of household member	Quality (daily and dinner): patterns of HEI-2010 component scores (i.e., total vegetable, greens/beans, total fruit, whole grains, dairy, total protein foods, seafood and plant proteins, fatty acid ratio, refined grains, SoFAAS)	Participants with higher frequency of "cooked dinner at home" [h]:<br>Daily pattern<br>↑ Probability to be classified into Class 4 ("Healthy US with high vegetable")<br>Dinner pattern<br>↑ Probability to be classified into Classes 2("SAD dinner with high Na"), 4 ("Healthy US dinner with high vegetable"), and 5 ("Healthy Mediterranean-style dinner")
Wolfson J, 2020[11]	NHANES, 2007-2010	National representative sample (≥20y), n 8668 (NA)	No	Multivariable linear regression stratified by income status for association; all covariates were adjusted regardless they were significant or not	Age, sex; total energy, race/ethnicity, country of birth, education, employment status, marital status, household size; SNAP participation, WIC participation, food security status; supplementary analyses further adjusted number of fast-foods, ready-to-eat, and frozen meals	Quality (daily): HEI-2015	Higher frequency of "cooking dinner at home":<br>↑ total HEI-2015 score

Wolfson J, 2015a[12]	NHANES, 2007-2010	National representative sample (≥20y), n 9560 (NA)	No	Multivariate logistic model for estimating the relationship between SNAP status and the probability of consuming any fruit and vegetables; generalized linear models used to examine the relationship between cooking frequency, SNAP status and fruit and vegetable intake	Gender, race/ethnicity, age, education, marital status, employment status, country of birth, household size, household food security; stratified by SNAP status	Intake (quantity, daily): total fruits/vegetables (including raw, fresh, frozen, canned, dried and pickled), and fresh fruits/vegetable (including only raw or cooked from raw) (white potatoes and sauces were excluded from vegetable group)	Higher frequency of "household cooking" : ↑/↔ Prevalence of consuming fresh vegetables ↑/↓ Total and fresh fruit intakes ↑/↔ Total and fresh vegetables intakes(results depended on SNAP status)
Wolfson J, 2015b[13]	NHANES, 2007-2010	National representative sample (≥20y), n 9569 (NA)	No	Multivariate linear model	Body weight, gender, race/ethnicity, age, education, marital status, employment status, country of birth, household size, household food security, day of the week, and weight-loss intention	Intake (quantity, daily): total energy, protein, fiber, CHO, fat and sugar	"High" compared with "medium" and "low" frequency of "cooking":<br>↓ Total energy, CHO (than "low" only), sugar (than "low" only), and fat (than "low" only)<br>↑ Fiber<br>↔ Protein
Taillie L, 2017[14]	NHANES, 2007-2010	Adults meeting the eligibility for participating SNAP (i.e., household income <130% FPL) (19-65y), n 2578 (NA)	No	Multivariable-adjusted survey weighted regression	Age (age and age squared), gender, race/ethnicity, education, quartiles of family income as percentage of FPL, survey year, marital status, physical activity; additionally adjusted fast-food and the interaction of fast-food with SNAP status	Intake (kilocalorie or weight, daily): total energy, and energy intakes from SoFAS, added sugar, solid fat, energy density of foods, SSBs, fruit (excluding juice), and non-starchy vegetables	"Higher" compared with "lower" frequency of "home-cooked dinner":<br>↓ SoFAS, solid fat, energy density of foods, SSBs<br>↔ Total energy, added sugar, fruit, and non-starchy vegetables

Tiwari A, 2017[15]	SOS, 2011-2013	Regional representative sample of English-speaking, primary food shoppers of the household, without any mobility issue in Seattle area (21-55y), n 437 (73%)	No	Multivariable linear regression	Age, gender, race/ethnicity, marital status, household size, employment status, education, household income	Quality (daily): HEI-2010 & 2005, and components of HEI-2010 (i.e., empty calories, Na, refined grains, fatty acids, seafood and plants, dairy, whole grains, greens and beans, total vegetables, whole fruit, total fruit)	"High" compared with "low" frequency of "cooking dinner at home":<br>↑ Total scores of HEI-2010 & 2005, and scores of empty calories, fatty acids, seafood & plant protein, greens & beans, total vegetables, whole fruit, and total fruit<br>↔ Na, refined grains, total proteins, dairy, and whole grains
Lam M, 2017[16]	NDNS, 2008-2009	National representative sample (≥19y), n 509 (NA)	No	Multivariate linear regression	Gender, age, occupation, household composition	Intake (contribution of energy, daily): ultra-processed foods	"Cooking main meal 5+ time per week" compared with "less often": ↓ % Energy intake from ultra-processed foods
Ozawa K, 2018 (in Japanese)[17]	*Saitama Kenmin Kenko Eiyou Chōsa* (nutritional survey in Saitama Prefecture), 2011	Urban middle-aged residents living in Saitama Prefecture (30-59y), n 384 (51%)	Yes	Multivariate logistic regression to examine the likelihood of consuming ≥ 300g/d versus <300g/d vegetables	Age, household composition, household income. Analysis stratified by sex	Intake (quantity, daily): vegetables (cut point: <300 and ≥300 g/d)	"Almost everyday" compared with "other than almost everyday":<br>↑ Probability of eating >300g vegetables (women only)
McGowan L, 2016[18]	(NR)	Nationally representative sample, who prepared a main meal at least 1 or 2x/wk (20-60y), n 1049 (NA)	No	Hierarchical multiple regression	Model 1: age, gender, education, SES; model 2: nutrition knowledge; model 3: food and health consciousness, cooking identity, food neophilia; model 4: cooking and food skill abilities	Quality (daily): Eating Choice Index (ECI)<br><br>Intake (score as proportion to standard portion size, daily): fat and fiber	Increased frequency of "meal preparation":<br>↑ ECI score, intakes of fat and fiber
Laska M, 2015 [19]	Student Health and Wellness survey, 2010	University/ community college students (21.4y), n 1013 (NR)	Yes/No[i]	Multivariable linear regression	Mutually adjusted for food preparation, time to prepare dinner; meal routines, media during meals, campus purchasing, time to eat dinner; age, gender, 2-/4-yr school, race/ethnicity, relationship status	Intake (cup-equivalent, daily): fruit and vegetables, fast foods, SSBs	Increased frequency of both "prepare meal at home" and "prepare own dinner"<br>↔ Fast-food, and SSBs<br>Increased frequency of "prepare meal at home"<br>↑ Fruit and vegetable intake<br>Increased frequency of "prepare own dinner" only<br>↓ Fruit and vegetable intake (for "prepare own dinner")

Bassul C, 2020[20]	Pre-schoolers health study, 2016-2017	Pre-school children from different geo-socioeconomic areas of Dublin, Ireland (3-5y), n 332 (50%)	Yes	Multiple logistic regression (<1 and ≥ 1x/wk for confectionary/SSBs, <1 and ≥1x/d for FV)	Adjusted all variables (42 in total) significant in the unadjusted model by target foods	Intake (serving, daily): fruit, vegetables (cut point: 1 serving for fruit and vegetables), confectionary or SSBs (cut point: 1x/wk)	Higher frequency of "parents prepare meals at home":<br>↔ Probabilities of eating ≥1x/d of vegetables, fruit, or ≥1x/wk of confectionary or SSBs
Mills S, 2017[21]	Fenland, 2005-2015	Adults born between 1950 and 1975 from general practice list in Cambridgeshire, UK (29-64y), n 11396 (27%)	No	Linear regression	Sex, age, smoking status, physical activity, working status, working overtime, and years of full education	Quality (daily): MDS and DASH<br><br>Intake (quantity, daily): fruit and vegetable	Higher frequency of "consuming home cooked meals":<br>↑ DASH score, MDS, and intakes of fruit and vegetable
Zong G, 2016[22]	NHS, 1976; HPFS, 1986	Middle-aged and older US health professionals (40-75y) and registered nurses (30-55y), women: n 58051 (NA); men: n 41676 (NA)	No	Descriptive, not statistical test	(NA)	Quality (daily): AHEI<br><br>Intake (serving, daily): total energy, fruits, vegetables, red meats, processed meats, dairy products, carbonated beverages, coffee consumption, French fries, whole grains, trans fatty acids, P/S ratio, Na	(Quality and intake were presented by frequency categories of "consuming meals prepared at home" but no statistical test was conducted)
Appelhans B, 2014[23]	HECS, (NR)	Children from low-income urban household in Chicago (6-13y), n 103 (NR)	No	Spearman correlation	(NA)	Intake (frequency, weekly): fast food, regular soda, diet soda, sports drinks, other sweetened beverages, milk consumed as a beverage, coffee drinks, 100% fruit juice, fruit, lettuce salad, vegetables (expect salad and dried potatoes).	Increased frequency of "home-prepared dinner consumption":<br>↓ Fast food, other sweetened beverages, and coffee drinks<br>↑ Vegetables (except salad and fried potatoes)<br>↔ Regular soda, diet soda, sports drinks, milk consumed as a beverage, 100% fruit juice, fruit
Erinosho T, 2012[24]	(NR), 2005-2006	Children who attend child-care centers in New York city (3-5y), n 200 (NA)	Yes	Binary logistic regression	Parent's ethnicity, and highest level of education completed	Intake (frequency, daily or weekly): fruits, 100% fruit juice, vegetables (cut point: 1x/d), French fries, soft drinks (cut point: 1x/wk), fruit drinks, desserts, and snacks (cut point: 3x/wk)	Children's dietary intakes at home "daily" compared with "≤6d/wk":<br>↑ Probability of eating ≥1x/d of fruit, and vegetables<br>↓ ≥ 3x/wk of snacks<br>↔ ≥1x/d of 100% fruit juice, ≥1x/wk of French fries and soft drinks, and ≥ 3x/wk of fruit drinks and desserts

Overcash F, 2020[25]	FLASHE, 2014	Adolescent drawn according to the US population (12-17y), n 1657 (NA)	No	Multivariable logistic regression	Age, sex, weight status, race, annual household income, parental education, 4 food groups	Intake (frequency, daily): fruit and vegetables (i.e., 100% fruit juice, fruit, green salad, other non-fried vegetables, cooked beans and other potatoes), junk food (candy/chocolate, cookies/cake, potato chips, fried potatoes, and frozen dessert), convenience food (fried potatoes, fried chicken, pizza, tacos, burgers, and heat-and-serve foods), SSB (soda, energy drinks, sweetened fruit drinks and sport drinks) (cut point: 1x/d)	"Any" compared with "none" of evening meal eaten at home was comprised of "cooked from scratch meals":<br>↑ Fruits and vegetables, and junk food<br>↔ Convenience foods and SSBs
Crawford D, 2006[26]	(NR)	Women from 45 Melbourne suburbs with different regional SES (18-65y), n 1580 (50%)	Yes	Multivariate logistic regression to examine the likelihood of consuming ≥ 2 daily servings of fruit and ≥2 daily servings of vegetables	Area-level SES, age, educational status (a measure of person SES), marital status, and the presence of children	Intake (serving, daily): fruits and vegetables (cut point: 2 servings/d)	Higher frequency of "eating meals that are prepared/cooked and eaten at home":<br>↑ Probability of eating ≥2 daily servings of fruit, and vegetables
Martins C, 2021[27]	(NR), 2015	Children from 1-4th grade of elementary school whose students are of parents working in the industry (NR), n 551 (49%)	Yes	Linear regression (HC as patterns extracted using principal component analysis)	Sex, age, race, marital status, education, employment status, number of children in the household and family income per capita	Intake (contribution of energy, dinner): ultra-processed foods	Higher adherence to "Healthy cooking" pattern compared with other patterns (i.e., "usual" and "convenience" cooking):<br>↓ % Energy intake from ultra-processed foods
Blake C, 2011[28]	(NA), 2006	Employed parents from low- to moderate-income urban area (23-56y), n 50 (NR)*	Yes	ANOVA （HC as patterns extracted using cluster analysis)	(NA)	Quality (daily): HEI-2005 and its components (i.e., total fruit, whole fruit, total vegetable, dark-green and orange vegetables, total grains, whole grains, milk, meat and bean, SFA, Na, SoFAAS, and oil)	"Home cooking" cluster compared with other clusters (i.e., "Individualized eating" and "Missing meals")[j]:<br>↑ Scores of dark-green and orange vegetables, and milk<br>↔ Scores of total HEI, total fruit, whole fruit, total vegetable, total grains, whole grains, meat and bean, saturated fat, Na, oil, and SoFAAS
Yoshiba K, 2015 (in Japanese)[29]	(NR), 2013	Elderly who living alone in Saitama Prefecture (65-89y), n 1043 (60%)	No	Multivariable logistic regression stratified by sex (covariates selected using stepwise method)	Potential covariates: Age, income, subjective economic status, education, and frailty status	Quality (daily): diversity [k] (cut point: 3)	"Prepared by myself" compared with summation of frequencies of other categories:<br>↑ Probability of adherence to higher dietary diversity (dinner for women)

Appelhans B, 2012[30]	"Dieting and decision making" study, (NR)	Overweight and obese women (18-45y); n 78 (83%)	Yes	ANOVA and linear mixed regression for energy intake per food item and per gram of food intake across home-prepared, away-from-home, and ready-to-eat	Age, BMI, education, income, ethnicity/race, marital status, perceived taste, and whether the food was consumed as part of a meal or as a snack	Intake (daily): per food item energy intake and per food item energy density	"Home-prepared" compared with "ready-to-eat" foods:<br>↑ Per food energy intake<br>↔ Per food energy density<br>"Home-prepared" compared to "away-from-home" foods:<br>↓ Per food energy intake, and per food energy density
Guthrie J, 2002[31]	NFCS 1977-1978, CFSII 1994-1996	National representative sample (≥2y); (NR) (NA)	Yes	t-test for intake difference between locations	Stratified by age (i.e., 2-17, ≥ 18); adjusted for sampling weights	Intake (energy contribution or energy-adjusted weight, daily)[1]: total fat, SFA, Na, fiber, Ca, and Fe	"Home" compared with "away" intake:<br>Adults<br>↓ Total fat, SFA, cholesterol, and Na<br>↑ Fiber, Ca, and Fe<br>Children<br>↓ Total fat, and SFA<br>↑ Cholesterol, fiber, Ca, and Fe<br>↔ Na
Smith L, 2013[32]	NFCS 1965-1966,1977-1978; CSFII 1989-1991, 1994-1996; NHANES 2003-2004, 2007-2008	National representative sample (19-60y); n 3138-12935 (NA)	Yes	t-tests were conducted to test differences between ratios of the means for each survey year to the previous year	(Participants were adjusted to be nationally representative)	Intake (contribution, daily): energy	Energy intake from "home food source" decreased from 1965 (~90%) to 2008 (~70%)
Smith T, 2019[33]	CSFII 1994-96, NHANES 2007-10	National representative sample (20-79y); n 5058-7662 (NA)	Yes	t-test between surveys	(NA)	Intake (contribution, daily): energy	↑ % Energy intake from "home" in 2007-2010 compared with 1994-1996 (only in Day 2)
Kwon Y, 2018[34]	KNHANES, 2010-2011	National representative children and adolescents (7-18y), n 2538 (NA)	Yes	Multiple logistic regression analysis for identifying factors affecting intake of ≥400g/d fruit and vegetables	Energy intake	Intake (quantity, daily): total food, and fruit and vegetables (exclude vegetable and fruit juice, starchy vegetables like potatoes and sweet potatoes) (cut point: 400g/d)	"Only home" compared with other categories:<br>↔ Probability of eating ≥400g/d of Fruits and vegetables (only higher than "only institution")

Kim S, 2018[35]	KNHANES, 2013-2014	Male workers drawn from national representative survey (19-64y), n 1634 (NA)	Yes	Chi-square test or ANCOVA	Age, occupation, education, household income, marital status and energy intake from dinner	Intake (energy contribution or weight, dinner)[m]: total energy, energy from dinner, macro- (i.e., CHO, protein, fat, SFA, MUFA, PUFA, and n-3 and n-6 fatty acids) and micronutrients (vitamins A, B1, B2 and C, niacin, Ca, P, Fe, Na, K)	"Eating at home" compared with "eating out of home":<br>↑ CHO (g), CHO (% energy), Fe, and vitamin B1<br>↓ Energy from dinner, protein (% energy), fat (g), fat (% energy), P, Na, K, SFA (g), PUFA (g), and n-6 fatty acids (g)<br>↔ Total energy, protein (g), Ca, vitamins A, B2 and C, niacin, MUFA (g), and n-3 fatty acids (g)
Nishi S, 2018[36]	CCHS, 2004-2005	National representative sample (≥2y), n 20402 (NA)	Yes	(NR)	(NA)	Intake (contribution, daily): total energy, Na, SFA, and added sugar intake	"Home" is the main source of energy (51-62%), SFA (50-61%), added sugar (50-65%), and Na (73-93%) intakes*
Wellard-Cole L, 2021[37]	MYMeals, 2016	Young Australians from New South Wales regional SES strata (18-30y), n 1001 (NR)[o]	Yes	(NR)	(NR)	Intake (contribution, daily): energy, protein, total fat, SFA, CHO, total sugar, Na	Foods from "home" consisted of 57% total energy, 58% protein, 57% total fat, 57% SFA, 57% CHO, 60% total sugar, and 52% Na[n]
McLaughlin C, 2003[38]	(NR), 1996-1997	Women using charitable food assistance programs in Toronto with at least one child <15y (19-49y); n 153 (68%)	Yes	Least-squares regression	Energy intake	Intake (energy-adjusted weight, daily): food groups (i.e., Fruits and vegetables, grain products, meat and alternates, milk products), and nutrients (i.e., CHO, protein, fat, vitamins C and A, folate, Fe, Mg, Zn, and Ca)	Increased both complexity of food preparation and frequency of food preparation from scratch:<br>↑ Fruit and vegetables, grain products, meat and alternatives, vitamin C, folate, Mg, Zn<br>↔ Milk products, other foods, and Ca<br>Increased complexity of food preparation only:<br>↑ CHO, and vitamin A<br>↓ Fat<br>Increased of frequency of food preparation from scratch only:<br>↑ Protein, and Fe
Astbury C, 2019a[39]	NDNS, 2008-2016	National representative sample (≥19y); n 6364 (NA)	Yes	Logistic regression to determine the association between proportion of energy from HPF and DASH accordance in the top quintile	Age, sex, ethnicity, presence of children in the household, occupation, education, household income	Quality (daily): DASH	Increased home-prepared food intake: ↑ DASH adherence to the top quintile

Astbury C, 2019b[40]	NDNS, 2008-2016	Individuals from the highest tertile of DASH score with home preparation from either the highest or lowest tertile (≥19y); n 1063 (NA)	Yes	Difference between groups was tested using linear or logistic regression	Age, sex, ethnicity, with children aged <16, education, income and occupation	Quality (daily): DASH and adherence to dietary guidelines (i.e., fat [<35% energy], SFA [<11% energy], protein [45-56g], sugar [<11% energy], Na [<1600mg], CHO)<br><br>Intake (quantity): low-fat dairy, whole grain, fruit, vegetables, nuts & legumes, Na, sugars, red & processed meats	"High" compared with "low" home preparation:<br>↔ DASH score, adherence to CHO, and intakes of whole grain, vegetables and nuts & legumes<br>↓ Adherence to fat, and intakes of low-fat dairy, fruit, Na and sugars<br>↑ Adherences to SFA, protein, sugar and Na, and intake of red & processed meats

A-CHILD, Adachi Child Health Impact of Living Difficulty project; AHEI, Alternative Healthy Eating Index; BDHQ, brief-type self-administered diet history questionnaire; BHCK, B' More Healthy Communities for Kids; CCHS, Canadian Community Health Survey; CHO, carbohydrate; CSFII, Continuing Survey of Food Intakes by Individuals; DA, dietary assessment; DASH, Dietary Approaches to Stop Hypertension; DISTANCE, Diabetes Study of Northern California; DR, dietary recall; FFQ, food frequency questionnaire; FLASHE, Family Life, Activity, Sun, Health, and Eating; FPL, Federal Poverty Line in the US; HC, home cooking; HECS, Home Environment Comparison Study; HEI, Healthy Eating Index; HPFS, Health Professionals Follow-up Study; KNHANES, Korea National Health and Nutrition Examination Survey; MDS, Mediterranean Diet Score; MUFA, monounsaturated fat; MYMeals, Measuring Young adults' Meals; NA, not available; NDNS, National Diet and Nutrition Survey; NFCS, Nationwide Food Consumption Survey; NHANES, National Health and Nutrition Examination Survey; NHS, Nurses' Health Study; NR, not reported; P/S, polyunsaturated to saturated fatty acids; PUFA, polyunsaturated fat; SES, socio-economic status; SFA, saturated fat; SNAP, Supplemental Nutrition Assistance Program; SoFAAS, solid fat, alcohol, added sugar; SoFAS, solid fat and added sugar; SOS, Seattle Obesity Study; SSB, sugar-sweetened beverages.

[a] For participation rate, "NA" refers to studies either used national representative datasets or cited external studies; "NR" means no information provided. [b] Assessed based on the concurrence of meal occasions assessed for "home cooking" and dietary variables. If methods for assessing "home cooking" specified a meal occasion (e.g., dinner), and dietary variables also assessed for that meal occasion, then "yes"; if methods for assessing "home cooking" did not specify a meal occasion, and dietary variables studied based on daily intake, then "yes". Otherwise, "no". [c] Only included items that used for investigating the relationship with "home cooking". [d] If not otherwise indicated, "↑", "↓", and "↔" indicate "significant positive relationship", "significant negative relationship" and "null relationship", respectively, corresponding to an increased magnitude of the indicator of "home cooking". [e] Percentages of meals children consumed included the target foods. [f] Total energy was assessed as crude quantity (kcal/day). [g] Not based on statistical test. Eating vegetable twice a day (prevalence): "high" (45.4%), "medium" (20.6%), "low" (23.8%). [h] Daily patterns identified: Class 1 ("standard American diet [SAD]", ref): lowest scores of green/beans, whole grains, and SoFAAS among all the patterns; Class 2 ("SAD with low Na"): Class 1 but lower scores of protein foods and refined grains, and higher scores of dairy and Na; Class 3 ("Healthy US"): higher scores of vegetable, seafood, fatty acid ratio, lower scores of Na, and refined grains than Class 1; Class 4 ("Healthy US with high vegetable"): Class 3 with the highest score of greens/beans among all patterns; Class 5 ("Healthy US with low Na"): Class 3 but higher scores of green/beans, whole grains and Na. Dinner patterns identified: Class 1 ("SAD dinner", ref): lowest scores of vegetable, green/beans, fatty acid ratio, refined grains and SoFAAS, highest dairy among all the patterns; Class 2 ("SAD dinner with high Na"): Class 1 but higher score of protein foods, and lower score of Na; Class 3 ("SAD dinner with high seafood"): Class 1 with higher scores of protein foods and seafood, and lower score of Na; Class 4 ("Healthy US dinner with high vegetable"): highest scores of vegetable, greens/beans, and lowest score of Na among all patterns; Class 5 ("Healthy Mediterranean-style dinner"): Class 4 but higher score of seafood; Class 6 ("Healthy US dinner with low Na"): Class 4 with lower scores of greens/beans and protein foods, and higher score of Na. [i] Validated methods used for assessing intakes of fast-food, and fruits and vegetables, but non-validated method for sugar-sweetened beverages. [j] n 56 (68%) for all participants included; n 50 for participants included in the analysis of the relationship between HC and dietary variables. [k] Weekly consumption (as binary, namely "everyday" and "not everyday") for 10 food groups (i.e., meats, fish, eggs, dairy, legumes, dark green/orange vegetables, seaweed, fruits, tubers, fat/oils) with a total score of 10. [l] Adjusted to the energy intake by location. [m] Energy assessed for both daily and dinner, and dinner for other nutrients. Both crude and energy-adjusted quantities for CHO, protein; only crude quantity fat, Ca, P, Fe, Na, K, Vitamins A, B1, B2 and C, niacin, SFA, MUFA, PUFA, n-3 and n-6 fatty acids. [n] Results shown here are those for "acceptable" energy reporters (table 5 in the primary study). [o] Enrolment was programmed to allow researchers to assess progress in meeting quotas for all the designated demographic groups required with respect to age group, sex, SES and geographic location. Once a quota was filled, no further participants were recruited to that group.

Table S4. Methods for assessing "home cooking".

First author, year of publication	Related questions (response categories, if applicable) or classification procedures
	**Perception-dependent**
Gustat J, 2017[1]	How many times they prepared meals from scratch at home per day [response category unknown]
Hanson A, 2019[2]	How often per week participants prepared meals from basic ingredients such as combining ground beef, tomato sauce, cheese, and noodles to make lasagna. (Daily, 4-6, 2-3, 1, <1x/wk, never)
Fertig A, 2019[3]	Choices of the following descriptors that best characterized how the meal was prepared: a) "fast food/take-out (eaten at home or at a restaurant);" b) "pre-prepared foods (e.g., macaroni and cheese, frozen meals) or purchased snacks (e.g., fruit snacks, chips, granola bars, cereal);" and/or c) "homemade/freshly prepared foods (include fresh fruits or vegetables here)." Based on this question, each meal was classified into four mutually exclusive categories:<br>1) fully home-cooked meals (respondent chose home-cooked foods only);<br>2) partly home-cooked meals (respondent chose home-cooked foods plus pre-prepared and/or restaurant foods);<br>3) restaurant meals (respondent chose fast food/take-out only, or fast food/take-out and pre-prepared foods); or<br>4) pre-prepared meals (respondent chose pre-prepared foods only).
Pachucki M, 2018[4]	How many meals per week do you eat that have been prepared at home (meaning food that has put together and cooked yourself (or by someone else in the household) and has not been pre-prepared/take out/fast food)' (0-7 x/wk) [separately asked for breakfast, lunch, and dinner]
Tani Y, 2020[5]; Tani Y, 2019[6]	[Over the past month] "How many times did you or someone else in your family cook meals at home?" (almost every day, 4-5, 2-3 days/wk, a few days/month, and rarely)
Sattler M, 2015	1) In the past 7 days, how often did a member of your household prepare food for you? (1, 2-3, 4-6x/wk, 1, and ≥2x/d)<br>2) In the past 7 days, how often did you prepare food for yourself or others (including making yourself lunch)? (1, 2-3, 4-6x/wk, 1, and ≥2x/d)
Saito A, 2019[8]	'During the past 1 month, how many times per week did you cook dinner at home for your family and for yourself? (0-7x/wk)
Farmer N, 2019[9]; Farmer N, 2020[10]; Wolfson J, 2020[11]; Wolfson J, 2015a[12]; Wolfson J, 2015b[13]; Taillie L, 2017[14]; Tiwari A, 2017[15]	During the past 7 days, how many times did you or someone else in your family cook food for dinner or supper at home?' (0-7x/wk)
Lam M, 2017[16]	How often do you prepare a main meal for yourself or others?' (never, only for special occasion, <1x/wk, 1-2d/wk, 3-4d/wk, 5-6d/wk, every day)
Ozawa K, 2018 (in Japanese)[17]	Do you prepare meals? ' [*Anata wa, shokujidukuri wo surukoto ga arimasuka?*] (5 choices from 'almost everyday' to 'almost never')
McGowan L, 2016[18]	Number of other meals they typically prepared or cooked in the home each aside from the main meal" (1–"preparing food or cooking typically only once a day" to 5–"preparing or cooking food on multiple occasion through out the day")
Laska M, 2015[19]	Number of days/wk of 1) prepared a meal at home, and 2) prepared their dinner (0-7x/wk)
Bassul C, 2020[20]	How many days per week meals were prepared at home (0-7d/wk)
Mills S, 2017[21]	When eating your main meal at home, how often do you usually eat home cooked meals?' (never or rarely; 1-2x/wk; 3-5x/wk; >5x/wk)
Zong G, 2016[22]	1) How often your midday meals were prepared at home; 2) How often your evening meals were prepared at home (NHS, never, 1-2, 3-4, 5-6, 7x/wk; HPFS, never, 1-2, 3-4, 5-7x/wk)
Appelhans B, 2014[23]	How many days per week does your child eat a dinner that was made at home by you or someone else?' (0-7 days)
Erinosho T, 2012[24]	Frequency of children's consuming meals prepared at home (every day, 4-6d/wk, 2-3d/wk, only on Sundays or 1d/wk, never)
Overcash F, 2020[25]	How many days in the past week the evening meal eaten at home was comprised of food cooked from scratch or a recipe and eaten at home (0-7d/wk)
Crawford D, 2006[26]	About how many times per week do you eat meals that are prepared/cooked and eaten at home? (Never, <1, 1, 2-3, 4-5, >6-7 meals/wk, NA) [separately asked for breakfast, lunch, and dinner]
Martins C, 2021[27]	Cooking pattern extracted using principal component analysis based on the following questions:<br>– Frequency of dinner preparation (never, almost never, 1-2x/wk, 3-4x/wk, 5-6x/wk, every day)<br>– Frequency of replacing dinner meal for a snack (never, almost never, 1-2x/wk, 3-4x/wk, 5-6x/wk, every day)<br>– Time dedicated to preparing dinner during the week (number of minutes)<br>– Receive help with dinner preparation (yes, no)

	– Do not cook to buy the ready meal (yes, no)<br>– Do not buy because do not know how to prepare (yes, no)<br>– To adapt recipes (yes, no)<br>– To cook extra food for freezing (yes, no)<br>– To do a shopping list (yes, no)<br>– To plan the shopping with the weekly meals in mind (yes, no)<br>– To use natural seasonings (yes, no)<br>– To use ready-made seasonings (yes, no)<br>– To use culinary techniques (yes or no each for the following items): sauteing, frying, grilling, oven-baking or roasting, stewing, boiling, steaming, pressure cook, microwaving to reheat, microwave to cook<br>– To feel confident in (yes or no each for the following items): following a simple recipe, seasoning meat using only natural seasonings, making a homemade tomato sauce using only tomatoes and natural seasonings, preparing a homemade soup, cooking beans in pressure cooker, grilling meat, preparing a simple homemade cake, baking homemade bread, preparing lunch or dinner by combining foods and spices already existing in the house without a recipe, cooking new foods you've never tried before
Blake C, 2011[28]	Cluster analysis of food-choice coping strategies based on the following domains (a total of 22 questions):<br>1) Food at home and away<br>– In a typical week:<br>• >5 of your family's main meals are home-cooked<br>• ≥ 1 of your family's main meals are from a fast-food restaurant<br>• ≥ 1 of your family's main meals are take-out foods<br>• ≥ 0.5 of your family's main meals are at a sit-down or buffet restaurant;<br>– On busy days, you eat a meal in the car;<br>– After work, you grab something quick to eat at a fast-food restaurant or convenience store<br>2) Missing meals<br>– Because of your job you miss eating meals with your family;<br>– Between work and family you miss eating breakfast;<br>– Because of your job, you miss eating lunch;<br>– You overeat later after missing a meal<br>3) Individualized meals<br>– On work days:<br>• The children eat first and adults eat later;<br>• Your family watches television during the main meal;<br>• Everyone in your family fixes something different for a main meal;<br>• You eat your main meal with all or most of your immediate family together (often)<br>4) Speeding up<br>– On workdays your family's main meal:<br>• Includes canned or frozen entrées or boxed mixes;<br>• Is something that is quick to prepare (often);<br>• You eat while you work;<br>• At work, you grab something quick to eat instead of a meal<br>5) Planning<br>– You pack a lunch to take to work;<br>– You keep food available at work for snacks and meals;<br>– Your family cooks enough to have leftovers (often);<br>– Your family cooks more on days off so there will be good meals ready for work nights
Yoshiba K, 2015 (in Japanese)[29]	How do you mainly eat for *breakfast/lunch/dinner*? [*chōshoku (hirushoku, yūshoku) wa omoni dōshiteimasuka*] (meal prepared by myself [*jibun de tsukutte taberu*]; take-out or eating in a restaurant [*omise de kattamono wo taberu (gaishoku fukumu)*]; almost not eating [*tabenaikoto ga ōi*]; other [*sonohoka*])
**Perception-independent**	
Appelhans B, 2012[30]	Home-prepared foods identified based on if it required at least a minimal level of preparation prior to consumption, including baking or heating, chopping, or blending/mixing with other ingredients. Examples of home-prepared foods included mixed dishes such as casseroles, homemade sandwiches, cooked vegetables, and microwaveable entrees
Guthrie J, 2002[31]	Home food is defined as food purchased at a retail store, such as a grocery store, convenience store, or supermarket;<br>foods not from home were those prepared at restaurants and other foodservice establishments, regardless of were eaten
Smith T, 2013[32]	'Home' food sources include any food that is designated as purchased from a store, convenience store, or grocery/deli;<br>food that were not from the home or the store were considered as away-from-home food sources, including schools, cafeterias, restaurants, and fast food
Smith T, 2019[33]	Food sources were defined by "where food was obtained, not where food was consumed". "Home food" indicates foods obtained from the following sources: store (e.g., grocery or supermarket); soup kitchen/shelter/food pantry; Meals on Wheels; community food programs; mail order purchase; grown or caught by your or some you known.<br>Foods not included were those obtained from avenue with table service, including bars, lounges, and taverns; fast food is obtained from quick-service venue (e.g., pizza place, street vendor, or vending machines); all other venues are included in an "other" category (e.g., gifts, fundraising sales, residential dining facilities, and cafeterias).
Kwon Y, 2018[34]	"Home meal" included meal prepared at home, lunch box prepared at home, or prepared by neighbors or relatives.<br>Patterns according to the place where food was cooked were defined as follows:<br>• "Only home meal": food prepared at home;<br>• "Only commercial location": food prepared in commercial locations (restaurants, fast food, snack bar and meals purchased

	outside the home: bread/cookie, street-stall/store, packed meal, or instant foods, including ramen and others);<br>• "Only institution": food prepared at public institutional cafeteria (school, industry, nursery/kkindergarten senior citizen center, free-meal service, or meals from religious organizations);<br>• "H+C+I": meals were prepared at home, commercial locations, and institutions;<br>• "H+C": meals prepared at home and commercial locations;<br>• "H+I": meals prepared at home and institutions;<br>• "C+I": meals prepared at commercial locations and institutions
Kim S, 2018[35]	Consumption of dinner was classified according to the place where dinner had been prepared: Dinner eaten "at home" included meals prepared at home and by relatives or neighbors; foods not included were commercially prepared meals or box meals purchased at restaurants or stores
Nishi S, 2018[36]	Food that participants ate had been prepared was asked: "Home" includes individual's home and someone else's home
Wellard-Cole L, 2021[37]	Participants were asked to choose from a dropdown menu to indicate the location which food was obtained.<br>"Home" includes all foods prepared inside the home.<br>Location outside the home were bakery or patisserie; coffee chain; cold drinks chain; fast food chain; other take-away shop; ice cream parlor/frozen yoghurt; pub (public hotel) or club; service station or convenience store; and independent cafe or restaurant.
McLaughlin C, 2003[38]	Development and validation of the equation used to classify HC<br>1) From the total 2,095 eating occasions, a randomly selected subsample of 150 eating occasions was used for developing the method. The 150 eating occasions were stratified by three levels ("high", "medium" and "low") of complexity in food preparation based on the presence of recipes, the number of foods in the recipes, the number of food reported but not designated as part of a recipe, the time of the day of the eating occasion (i.e., before, or afternoon);<br>2) Intake of each food at each eating occasion was assigned scores by an investigator based on the following techniques (1 score per technique): washing, subdivision and fraction, combining and mixing, heating, and the removal of heat;<br>3) For developing the predictive equation, the complexity scores for the 150 eating occasions were regressed on four variables: binary variable for the presence or absence of recipe, number of foods included in the recipe, number of foods not included in the recipe, and the time of consumption. Only parameters that showed statistical significance were included in the equation:<br>Food preparation complexity = (2.09*number of recipe foods) ± 0.09+(1.18*number of foods in the eating occasion that not included in a recipe) ± 0.21 (R2 = 0.78)<br>4) For validation, a second 150 eating occasions were independently selected based on the quartiles of the score assigned based on step 2) by another investigator. 75 eating occasions were selected from the first quartile, and 75 were selected from the bottom three quartiles. The correlations between the assigned and predicted scores were 0.87 and 0.92 for Pearson and Spearman, respectively.<br><br>Application of the developed equation<br>1) Using the developed equation to predict all eating occasions that included home as a location of food preparation (otherwise assigned zero);<br>2) Summation of the scores overall eating occasions for each participant;<br>3) Food preparation complexity was represented as the average over the 3-day score;<br>4) Food preparation from scratch was determined by the equation using a cut-off at 8 calculated from the equation of "food preparation complexity" by re-examining the data for each meal per participant (meal occasion with a recipe containing 4 ingredients had complexity score with a mean of 8.36 [SE 0.4]);<br>5) Average frequency of food preparation over the three days was calculated for each individual
Astbury C, 2019a[39]; Astbury C, 2019b[40]	'Home-prepared' foods were classified by EXCLUDING the follows:<br>1) Foods prepared and eaten outside the home (e.g., food eaten in a restaurant or café)<br>2) Foods prepared outside the home and eaten in the home (e.g., takeaway and delivery foods)<br>3) Foods eaten as purchased (e.g., crisps, sweets, granola bars, juice and soft drinks, store-bought sandwiches, prepared and whole pieces of fruit)<br>4) Foods requiring the application of heat or the addition of hot water but no other preparation (e.g., frozen and refrigerated ready meals, tinned soup, instant noodles, instant oats)<br>5) Foods involving the combination of several components by the participant, but each component required minimal preparation (e.g., a bowl of cereal, a ham or cheese sandwich) [for Astbury C, 2019(b) only]

Table S5. Quality assessment of the included studies.

First author, year of publication	Representativeness	Confounding	Methods of 'home cooking'	Methods of dietary variables	Concurrence of meal occasion	Data analysis	Total count			Overall
							"High"	"Moderate"	"Low"	
Gustat J, 2017[1]	Moderate	Moderate	Low	Low	High	High	2	2	2	**Low**
Hanson A, 2019[2]	Low	High	Low	Moderate	High	High	3	1	2	**Low**
Fertig A, 2019[3]	Low	High	Low	Low	High	High	3	0	3	**Low**
Pachucki M, 2018[4]	Low	High	Low	High	High	High	4	0	2	**Low**
Tani Y, 2020[5]	High	Low	Low	Low	High	Moderate	2	1	3	**Low**
Tani Y, 2019[6]	High	Low	Low	Low	High	Moderate	2	1	3	**Low**
Sattler M, 2015[7]	Low	Low	Low	Moderate	High	High	2	1	3	**Low**
Saito A, 2019[8]	Moderate	High	Low	Moderate	Moderate	High	2	3	1	**Low**
Farmer N, 2019[9]	High	High	Low	High	High	High	5	0	1	**Moderate**
Farmer N, 2020[10]	High	High	Low	High	High	High	5	0	1	**Moderate**
Wolfson J, 2020[11]	High	High	Low	High	Moderate	High	4	1	1	**Moderate**
Wolfson J, 2015a[12]	High	High	Low	High	Moderate	High	4	1	1	**Moderate**
Wolfson J, 2015b[13]	High	High	Low	High	Moderate	High	4	1	1	**Moderate**
Taillie L, 2017[14]	High	High	Low	High	Moderate	High	4	1	1	**Moderate**
Tiwari A, 2017[15]	Moderate	High	Low	Moderate	Moderate	High	2	3	1	**Moderate**
Lam M, 2017[16]	High	Low	Low	High	Moderate	High	3	1	2	**Low**
Ozawa K, 2018 (in Japanese)[17]	Low	Moderate	Low	High	High	High	3	1	2	**Low**
McGowan L, 2016[18]	High	High	Low	Moderate	Moderate	High	3	2	1	**Moderate**
Laska M, 2015[19]	Low	High	Low	Low	High	High	3	0	3	**Low**
Bassul C, 2020[20]	Low	High	Low	Low	High	High	3	0	3	**Low**
Mills S, 2017[21]	Low	Low	Low	Moderate	Moderate	High	1	2	3	**Low**
Zong G, 2016[22]	NA	Low	Low	Moderate	Moderate	Moderate	0	3	2	**Low**
Appelhans B, 2014[23]	Low	Low	Low	Low	Moderate	Moderate	1	2	3	**Low**
Erinosho T, 2012[24]	NA	Low	Low	Low	High	High	2	0	3	**Low**
Overcash F, 2020[25]	NA	Moderate	Low	Low	Moderate	High	1	2	2	**Low**
Crawford D, 2006[26]	Low	Low	Low	Low	High	High	2	0	4	**Low**
Martins C, 2021[27]	Low	High	High	High	High	High	5	0	1	**Moderate**
Blake C, 2011[28]	Moderate	Low	High	High	High	Moderate	3	2	1	**Moderate**
Yoshiba K, 2015 (in Japanese)[29]	Moderate	Low	Low	Low	Low	High	2	1	3	**Low**
Appelhans B, 2012[30]	High	High	Moderate	High	High	High	5	1	0	**Moderate**

Guthrie J, 2002[31]	High	NA	Moderate	High	High	High	4	1	0	**High**
Smith L, 2013[32]	High	NA	Moderate	High	High	High	4	1	0	**High**
Smith T, 2019[33]	High	NA	Moderate	High	High	High	4	1	0	**High**
Kwon Y, 2018[34]	High	Low	Moderate	High	High	High	4	2	0	**Moderate**
Kim S, 2018[35]	High	High	Moderate	High	High	High	5	1	0	**High**
Nishi S, 2018[36]	High	NA	Moderate	High	High	High	4	1	0	**High**
Wellard-Cole L, 2021[37]	High	Low	Moderate	High	High	High	4	1	1	**Moderate**
McLaughlin C, 2003[38]	Moderate	Low	High	High	High	High	4	1	1	**Moderate**
Astbury C, 2019a[39]	High	High	High	High	High	High	6	0	0	**High**
Astbury C, 2019b[40]	High	High	High	High	High	High	6	0	0	**High**

References

1. Gustat, J.; Lee, Y.-S.; O'Malley, K.; Luckett, B.; Myers, L.; Terrell, L.; Amoss, L.; Fitzgerald, E.; Stevenson, P.T.; Johnson, C.C. Personal Characteristics, Cooking at Home and Shopping Frequency Influence Consumption. *Prev Med Rep* **2017**, *6*, 104–110, doi:10.1016/j.pmedr.2017.02.007.
2. Hanson, A.J.; Kattelmann, K.K.; McCormack, L.A.; Zhou, W.; Brown, O.N.; Horacek, T.M.; Shelnutt, K.P.; Kidd, T.; Opoku-Acheampong, A.; Franzen-Castle, L.D.; et al. Cooking and Meal Planning as Predictors of Fruit and Vegetable Intake and BMI in First-Year College Students. *Int J Environ Res Public Health* **2019**, *16*, 2462, doi:10.3390/ijerph16142462.
3. Fertig, A.R.; Loth, K.A.; Trofholz, A.C.; Tate, A.D.; Miner, M.; Neumark-Sztainer, D.; Berge, J.M. Compared to Pre-Prepared Meals, Fully and Partly Home-Cooked Meals in Diverse Families with Young Children Are More Likely to Include Nutritious Ingredients. *J Acad Nutr Diet* **2019**, *119*, 818–830, doi:10.1016/j.jand.2018.12.006.
4. Pachucki, M.C.; Karter, A.J.; Adler, N.E.; Moffet, H.H.; Warton, E.M.; Schillinger, D.; O'Connell, B.H.; Laraia, B. Eating with Others and Meal Location Are Differentially Associated with Nutrient Intake by Sex: The Diabetes Study of Northern California (DISTANCE). *Appetite* **2018**, *127*, 203–213, doi:10.1016/j.appet.2018.03.020.
5. Tani, Y.; Fujiwara, T.; Kondo, K. Cooking Skills Related to Potential Benefits for Dietary Behaviors and Weight Status among Older Japanese Men and Women: A Cross-Sectional Study from the JAGES. *Int J Behav Nutr Phys Act* **2020**, *17*, 82, doi:10.1186/s12966-020-00986-9.
6. Tani, Y.; Fujiwara, T.; Doi, S.; Isumi, A. Home Cooking and Child Obesity in Japan: Results from the A-CHILD Study. *Nutrients* **2019**, *11*, 2859, doi:10.3390/nu11122859.
7. Sattler, M.; Hopkins, L.; Steeves, E.A.; Cristello, A.; Hurley, K.; McCloskey, M. Characteristics of Youth Food Preparation in Low-Income, African American Homes: Associations with Healthy Eating Index Scores. *Ecol Food Nutr* **2015**, *54*, 380–396, doi:10.1080/03670244.2014.1001982.
8. Saito, A.; Matsumoto, M.; Hyakutake, A.; Saito, M.; Okamoto, N.; Tsuji, M. The Frequency of Cooking Dinner at Home and Its Association with Nutrient Intake Adequacy among Married Young-to-Middle-Aged Japanese Women: The POTATO Study. *J Nutr Sci* **2019**, *8*, e14, doi:10.1017/jns.2019.9.
9. Farmer, N.; Wallen, G.R.; Yang, L.; Middleton, K.R.; Kazmi, N.; Powell-Wiley, T.M. Household Cooking Frequency of Dinner Among Non-Hispanic Black Adults Is Associated with Income and Employment, Perceived Diet Quality and Varied Objective Diet Quality, HEI (Healthy Eating Index): NHANES Analysis 2007–2010. *Nutrients* **2019**, *11*, 2057, doi:10.3390/nu11092057.
10. Farmer, N.; Lee, L.J.; Powell-Wiley, T.M.; Wallen, G.R. Cooking Frequency and Perception of Diet among US Adults Are Associated with US Healthy and Healthy Mediterranean-Style Dietary Related Classes: A Latent Class Profile Analysis. *Nutrients* **2020**, *12*, 3268, doi:10.3390/nu12113268.
11. Wolfson, J.A.; Leung, C.W.; Richardson, C.R. More Frequent Cooking at Home Is Associated with Higher Healthy Eating Index-2015 Score. *Public Health Nutr* **2020**, *23*, 2384–2394, doi:doi: 10.1017/S1368980019003549.
12. Wolfson, J.A.; Bleich, S.N. Fruit and Vegetable Consumption and Food Values: National Patterns in the

United States by Supplemental Nutrition Assistance Program Eligibility and Cooking Frequency. *Prev Med* **2015**, *76*, 1–7, doi:10.1016/j.ypmed.2015.03.019.

13. Wolfson, J.A.; Bleich, S.N. Is Cooking at Home Associated with Better Diet Quality or Weight-Loss Intention? *Public Health Nutr.* **2015**, *18*, 1397–1406, doi:10.1017/S1368980014001943.
14. Taillie, L.S.; Poti, J.M. Associations of Cooking With Dietary Intake and Obesity Among Supplemental Nutrition Assistance Program Participants. *Am J Prev Med* **2017**, *52*, S151–S160, doi:10.1016/j.amepre.2016.08.021.
15. Tiwari, A.; Aggarwal, A.; Tang, W.; Drewnowski, A. Cooking at Home: A Strategy to Comply With U.S. Dietary Guidelines at No Extra Cost. *Am J Prev Med* **2017**, *52*, 616–624, doi:10.1016/j.amepre.2017.01.017.
16. Lam, M.C.L.; Adams, J. Association between Home Food Preparation Skills and Behaviour, and Consumption of Ultra-Processed Foods: Cross-Sectional Analysis of the UK National Diet and Nutrition Survey (2008–2009). *Int J Behav Nutr Phys Act* **2017**, *14*, 68, doi:10.1186/s12966-017-0524-9.
17. Ozawa K.; Takemi Y.; Eto K.; Iwama N. Association of vegetable intake with dietary behaviors, attitudes, knowledge, and social support among the middle-aged Japanese population. *Japanese Journal of Public Health* **2018**, *65*, 589–601.
18. McGowan, L.; Pot, G.K.; Stephen, A.M.; Lavelle, F.; Spence, M.; Raats, M.; Hollywood, L.; McDowell, D.; McCloat, A.; Mooney, E.; et al. The Influence of Socio-Demographic, Psychological and Knowledge-Related Variables alongside Perceived Cooking and Food Skills Abilities in the Prediction of Diet Quality in Adults: A Nationally Representative Cross-Sectional Study. *Int J Behav Nutr Phys Act* **2016**, *13*, 111, doi:10.1186/s12966-016-0440-4.
19. Laska, M.N.; Hearst, M.O.; Lust, K.; Lytle, L.A.; Story, M. How We Eat What We Eat: Identifying Meal Routines and Practices Most Strongly Associated with Healthy and Unhealthy Dietary Factors among Young Adults. *Public Health Nutr* **2015**, *18*, 2135–2145, doi:10.1017/S1368980014002717.
20. Bassul, C.; Corish, C.A.; Kearney, J.M. Associations between the Home Environment, Feeding Practices and Children's Intakes of Fruit, Vegetables and Confectionary/Sugar-Sweetened Beverages. *Int J Environ Res Public Health* **2020**, *17*, 4837, doi:10.3390/ijerph17134837.
21. Mills, S.; Brown, H.; Wrieden, W.; White, M.; Beasley, J.M. Frequency of Eating Home Cooked Meals and Potential Benefits for Diet and Health: Cross-Sectional Analysis of a Population-Based Cohort Study. *Int J Behav Nutr Phys Act* **2017**, *14*, 109, doi:10.1186/s12966-017-0567-y.
22. Zong, G.; Eisenberg, D.M.; Hu, F.B.; Sun, Q. Consumption of Meals Prepared at Home and Risk of Type 2 Diabetes: An Analysis of Two Prospective Cohort Studies. *PLoS Med* **2016**, *13*, e1002052, doi:10.1371/journal.pmed.1002052.
23. Appelhans, B.M.; Waring, M.E.; Schneider, K.L.; Pagoto, S.L. Food Preparation Supplies Predict Children's Family Meal and Home-Prepared Dinner Consumption in Low-Income Households. *Appetite* **2014**, *76*, 1–8, doi:10.1016/j.appet.2014.01.008.
24. Erinosho, T.O.; Beth Dixon, L.; Young, C.; Brotman, L.M.; Hayman, L.L. Caregiver Food Behaviours Are Associated with Dietary Intakes of Children Outside the Child-Care Setting. *Public Health Nutr.* **2013**, *16*, 1263–1272, doi:10.1017/S136898001200345X.

25. Overcash, F.; Davey, C.; Zhang, Y.; Reicks, M. Evening Meal Types and Family Meal Characteristics: Associations with Demographic Characteristics and Food Intake among Adolescents. *Nutrients* **2020**, *12*, 886, doi:10.3390/nu12040886.
26. Crawford, D.; Ball, K.; Mishra, G.; Salmon, J.; Timperio, A. Which Food-Related Behaviours Are Associated with Healthier Intakes of Fruits and Vegetables among Women? *Public Health Nutr* **2007**, *10*, 256–265, doi:10.1017/S1368980007246798.
27. Martins, C.A.; Andrade, G.C.; Oliveira, M.F.B. de; Rauber, F.; Castro, I.R.R. de; Couto, M.T.; Levy, R.B. "Healthy", "Usual" and "Convenience" Cooking Practices Patterns: How Do They Influence Children's Food Consumption? *Appetite* **2021**, *158*, 105018, doi:10.1016/j.appet.2020.105018.
28. Blake, C.E.; Wethington, E.; Farrell, T.J.; Bisogni, C.A.; Devine, C.M. Behavioral Contexts, Food-Choice Coping Strategies, and Dietary Quality of a Multiethnic Sample of Employed Parents. *J Am Diet Assoc* **2011**, *111*, 401–407, doi:10.1016/j.jada.2010.11.012.
29. Yoshiba K.; Takemi Y.; Ishikawa M.; Yokoyama T.; Nakaya T.; Murayama N. Relationship between dietary diversity and food access among elderly living alone in Saitama Prefecture. *Japanese Journal of Public Health* **2015**, *62*, 707–718.
30. Appelhans, B.M.; Waring, M.E.; Schneider, K.L.; Pagoto, S.L.; DeBiasse, M.A.; Whited, M.C.; Lynch, E.B. Delay Discounting and Intake of Ready-to-Eat and Away-from-Home Foods in Overweight and Obese Women. *Appetite* **2012**, *59*, 576–584, doi:10.1016/j.appet.2012.07.009.
31. Guthrie, J.F.; Lin, B.-H.; Frazao, E. Role of Food Prepared Away from Home in the American Diet, 1977-78 versus 1994-96: Changes and Consequences. *J Nutr Educ Behav* **2002**, *34*, 140–150, doi:10.1016/s1499-4046(06)60083-3.
32. Smith, L.P.; Ng, S.W.; Popkin, B.M. Trends in US Home Food Preparation and Consumption: Analysis of National Nutrition Surveys and Time Use Studies from 1965–1966 to 2007–2008. *Nutr J* **2013**, *12*, doi:10.1186/1475-2891-12-45.
33. Smith, T.A.; Valizadeh, P.; Lin, B.-H.; Coats, E. What Is Driving Increases in Dietary Quality in the United States? *Food Policy* **2019**, *86*, 101720, doi:10.1016/j.foodpol.2019.05.003.
34. Kwon, Y.-S.; Kim, Y. Fruit and Vegetable Intake of Korean Children and Adolescents According to Cooking Location and Daily Meal: Study Based on 2010 and 2011 Korea National Health and Nutrition Examination Survey Data. *Asia Pac J Clin Nutr* **2018**, *27*, 217–230, doi:10.6133/apjcn.032017.09.
35. Kim, S.; Kang, M. Male Korean Workers Eating out at Dinner. *British Food Journal* **2018**, 12.
36. Nishi, S.; Jessri, M.; L'Abbé, M. Assessing the Dietary Habits of Canadians by Eating Location and Occasion: Findings from the Canadian Community Health Survey, Cycle 2.2. *Nutrients* **2018**, *10*, 682, doi:10.3390/nu10060682.
37. Wellard-Cole, L.; Davies, A.; Chen, J.; Jung, J.; Bente, K.B.; Kay, J.; Watson, W.L.; Hughes, C.; Rangan, A.; Yacef, K.; et al. The Contribution of Foods Prepared Outside the Home to the Diets of 18- to 30-Year-Old Australians: The MYMeals Study. *Nutrients* **2021**, *13*, 1761, doi:10.3390/nu13061761.
38. McLaughlin, C.; Tarasuk, V.; Kreiger, N. An Examination of At-Home Food Preparation Activity among Low-Income, Food-Insecure Women. *J Am Diet Assoc* **2003**, *103*, 1506–1512, doi:10.1016/j.jada.2003.08.022.

39. Astbury, C.C.; Penney, T.L.; Adams, J. Home-Prepared Food, Dietary Quality and Socio-Demographic Factors: A Cross-Sectional Analysis of the UK National Diet and Nutrition Survey 2008–16. *Int J Behav Nutr Phys Act* **2019**, *16*, 82, doi:10.1186/s12966-019-0846-x.
40. Astbury, C.C.; Penney, T.L.; Adams, J. Comparison of Individuals with Low versus High Consumption of Home-Prepared Food in a Group with Universally High Dietary Quality: A Cross-Sectional Analysis of the UK National Diet & Nutrition Survey (2008–2016). *Int J Behav Nutr Phys Act* **2019**, *16*, 9, doi:10.1186/s12966-019-0768-7.
